# Legumain deficiency halts atherogenesis by modulating T cell receptor signaling

**DOI:** 10.1111/acel.14391

**Published:** 2024-10-29

**Authors:** Xuying Xiang, Feng Zhang, Lei Nie, Xiaoqing Guo, Mengting Qin, Jiaojiao Chen, Dailiang Jiang, Zhentao Zhang, Ling Mao

**Affiliations:** ^1^ Department of Neurology Union Hospital, Tongji Medical College, Huazhong University of Science and Technology Wuhan China; ^2^ Department of Neurology Renmin Hospital of Wuhan University Wuhan China

**Keywords:** atherogenesis, Bcl‐2, legumain, T cell receptor

## Abstract

Atherosclerosis is an age‐related pathological process associated with elevated levels of legumain in plaques and plasma. However, the underlying mechanisms remain unclear. The aim of this study was to investigate the role of legumain in the progression of atherosclerotic plaques, with a particular focus on functional and phenotypic changes in CD4^+^ T cells. Apolipoprotein E‐deficient (*Apoe* ^−/−^) mice were crossed with legumain‐deficient (*Lgmn* ^−/−^) mice to generate *Lgmn* ^−/−^ *Apoe* ^−/−^ mice. CD4^+^ T cells accumulated in the atherosclerotic plaques of *Apoe* ^−/−^ mice fed a high‐fat diet. Deletion of legumain attenuated the deposition of CD4^+^ T cells in plaques and reduced the number of atherosclerotic lesions. The levels of CD4^+^ T cells in the blood, lymph nodes, and spleen were decreased in *Lgmn* ^−/−^ mice. Transcriptomic analysis revealed that the deletion of legumain decreased the differentiation, survival, and function of CD4^+^ memory T cells by suppressing the T cell receptor (TCR) signaling pathway. These changes are accompanied by the downregulation of the antiapoptotic protein B‐cell lymphoma 2 (Bcl‐2) and the reduced release of interleukin (IL)‐2 and interferon (IFN)‐γ. These results suggest that legumain deficiency may play a role in the development of atherosclerosis by impairing the survival, proliferation, and function of CD4^+^ T cells. Inhibition of legumain activity may be an innovative therapy for the treatment of atherosclerosis.

## Graphical abstract

Legumain deficiency impairs TCR signaling, CD4^+^ T cell survival and proliferation, and reduces the formation and function of effector T cells in *Apoe* ^−/−^ mice, conferring protection against atherosclerosis.

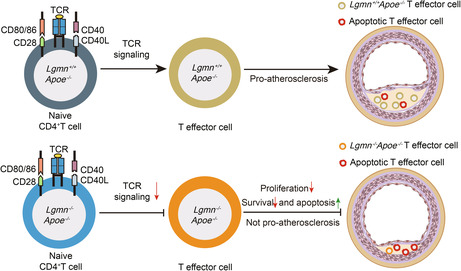

## 1 INTRODUCTION

Atherosclerosis is a chronic progressive inflammatory disease of the arterial wall and a major cause of aging‐related cardiovascular disease (Gisterå & Hansson, 2017). Recent technological advances, such as single‐cell RNA sequencing, cell lineage tracing, and mass spectrometry flow cytometry, have provided data revealing the critical regulatory role of T cells in plaque formation and development (Fernandez et al., 2019; McArdle et al., 2019; Wolf et al., 2020). CD4^+^ T helper cells drive proinflammatory responses by recognizing peptides derived from apolipoprotein B (ApoB), the core protein of low‐density lipoprotein (LDL) cholesterol particles, and producing cytokines under the endothelium of the arterial wall (Roy et al., 2022). This process is initiated by antigen recognition by the T cell receptor (TCR) and facilitated by co‐stimulatory signals, accompanied by T cell activation, proliferation, and differentiation (Carreno & Collins, 2002; Hwang et al., 2020). Although several clinical strategies are available for the treatment of atherosclerosis, these strategies focus primarily on controlling atherosclerotic risk factors and reducing hyperlipidemia and rarely work to reduce plaque (Sharma et al., 2020). Modulation of T cell immune function has been recognized as a clinically promising option (Weber et al., 2023). However, the exact mechanisms of T cell survival, proliferation, and differentiation in the atherosclerotic process remain to be elucidated.

Legumain is a lysosomal cysteine protease that specifically targets the C‐terminus of the asparagine residue of the substrate (Chen, Dando, et al., 1998; Chen, Rawlings, et al., 1998). The enzymatic activity of legumain is affected by pH, which means that homeostatic disturbances and subsequent lysosomal dysfunction may lead to an increase in legumain enzymatic activity, thereby contributing to disease progression (Brix et al., 2015; Li et al., 2003). Previous studies have shown increased expression of legumain in human carotid plaques and plasma. Legumain has also been observed in mouse models of atherosclerotic plaques (Lunde et al., 2017; Mattock et al., 2010). These findings suggest that legumain may be involved in the pathogenesis of atherosclerosis. Legumain has also been reported to promote atherosclerotic plaque instability by degrading extracellular matrix components through the activation of matrix metalloproteinase‐2 (Matsumura et al., 2005). Since legumain has an integrin binding motif, it can bind to integrin αVβ3 and block its downstream signaling (Pan et al., 2022). In addition to the above studies focusing on the function of legumain in macrophages, legumain has recently been reported to be highly expressed in T cells. Moreover, the level of legumain in T cells can be further increased by co‐stimulation (He et al., 2024). As a lysosomal enzyme, legumain may regulate the innate immune response by processing and activating lysosomal or endosomal nucleic acid‐sensing toll‐like receptors (TLRs) (Kim et al., 2021). Although legumain has been shown to accelerate major histocompatibility complex (MHC) class II antigen presentation, no difference in invariant chain processing or maturation of class II MHC products was observed in legumain‐deficient mice (Maehr et al., 2005; Manoury et al., 1998). In addition, the inhibition of legumain enzymatic activity has been shown to block cathepsin L double‐strand maturation and reduce C3 production, and increased legumain activity in human T cells is associated with increased interferon (IFN)‐γ production (Freeley et al., 2018). The transcription factor Foxp3, which plays a critical role in the development of regulatory T cells, can also be cleaved by legumain (Stathopoulou et al., 2018). These findings suggest that legumain may play a key role in regulating the immune response involving T cells. However, the role and function of legumain in T cells in cardiovascular disease, particularly in the progression of atherosclerosis, remain unclear.

In the present study, we investigated the effects of legumain knockout on T cell deposition in plaques and determined the mechanism by which legumain regulates T‐cell function during atherogenesis. We found that legumain deficiency inhibits plaque progression by regulating the TCR signaling pathway. These results suggest that legumain may be a therapeutic target for atherosclerosis.

## 2 RESULTS

### 2.1 Legumain deficiency halts the progression of atherosclerosis

**FIGURE 1:**
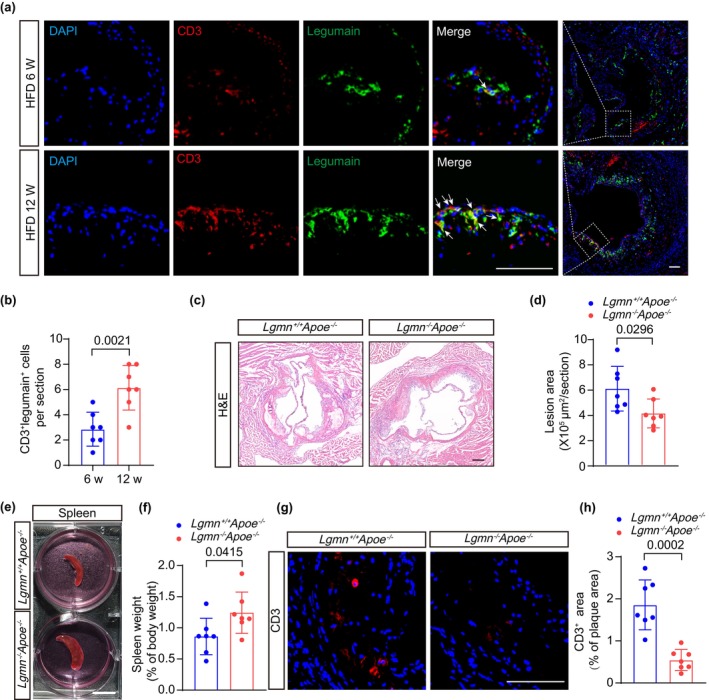
Legumain deficiency attenuates atherosclerosis in *Apoe* ^*−/−*^ mice. (a, b) Representative confocal microscopy images showing immunostaining for CD3 (red) and legumain (green) in aortic root plaques from *Apoe* ^−/−^ mice fed a high‐fat diet (HFD) for 6 or 12 weeks (W). Arrows indicate co‐localization of T cells (CD3) and legumain. Scale bar, 100 μm. The number of legumain‐positive T cells per section was quantified (*n* = 7 mice per group). (c, d) Representative hematoxylin and eosin (H&E)‐stained images of mouse aortic root plaques from *Lgmn* ^+/+^ *Apoe* ^−/−^ mice and *Lgmn* ^−/−^ *Apoe* ^−/−^ mice. The lesion area in the mouse aortic root was quantified (*n* = 7 mice per group). Scale bar, 200 μm. (e) Representative image of spleens from *Lgmn* ^+/+^ *Apoe* ^−/−^ mice and *Lgmn* ^−/−^ *Apoe* ^−/−^ mice. Scale bar, 1 cm. (f) Spleen weight/body weight in *Lgmn* ^+/+^ *Apoe* ^−/−^ mice and *Lgmn* ^−/−^ *Apoe* ^−/−^ mice (*n* = 7 mice per group). (g, h) Representative immunofluorescence images showing CD3‐stained aortic root sections from *Lgmn* ^+/+^ *Apoe* ^−/−^ mice and *Lgmn* ^−/−^ *Apoe* ^−/−^ mice (*n* = 7 mice per group). Scale bar, 100 μm. The ratio of CD3‐positive area to plaque area from each group was quantified (*n* = 7 mice per group). HFD, high‐fat diet; W, weeks. Data information: The exact *p* value is specified. The *p* value was determined by unpaired two‐tailed Student's *t* test (b, d, f, and h).

We first investigated the expression of legumain within T cells in the atherosclerotic plaques of apolipoprotein E‐deficient (*Apoe* ^−/−^) mice fed a high‐fat diet (HFD) for 6 or 12 weeks (Figure 1a). Immunofluorescence staining of atherosclerotic plaques in the aortic roots of *Apoe* ^−/−^ mice revealed that legumain colocalized with CD3‐positive T cells in the shoulder of the plaques and that the number of CD3^+^legumain^+^ T cells increased with prolonged HFD feeding (Figure 1b).

To investigate the role of legumain in atherosclerotic plaque formation, we crossed *Lgmn* ^−/−^ mice with *Apoe* ^−/−^ mice to generate *Lgmn* ^−/−^ *Apoe* ^−/−^ mice. *Lgmn* ^−/−^ *Apoe* ^−/−^ mice and littermate *Lgmn* ^+/+^ *Apoe* ^−/−^ control mice were fed HFD for 12 weeks. High‐fat feeding significantly increased body weight and plasma total cholesterol levels in both groups of animals. There were no statistically significant differences in body weight, lipid levels, or blood white blood cell counts between *Lgmn* ^*−/−*^ *Apoe* ^*−/−*^ mice and *Lgmn* ^+/+^ *Apoe* ^−/−^ mice, but decreased red blood cell and hemoglobin levels were observed in legumain‐deficient mice (Figure S1A, Table S1). Hematoxylin and eosin (H&E) staining of the aortic roots revealed a significant reduction in plaque burden (0.67‐fold) in *Lgmn* ^−/−^ *Apoe* ^−/−^ mice compared with *Lgmn* ^+/+^ *Apoe* ^−/−^ mice (Figure 1c,d). Surprisingly, the weights of the spleens of *Lgmn* ^−/−^ *Apoe* ^−/−^ mice were dramatically greater than those of *Lgmn* ^+/+^ *Apoe* ^−/−^ control mice (Figure 1e,f). In addition, the weight of *Lgmn* ^+/+^ *Apoe* ^−/−^ spleens was similar to that of the control group (C57BL/6) (Figure S1B,C). However, the weight of the thymus did not differ between the two groups (Figure S1D,E).

Changes in spleen weight suggest that legumain deficiency may affect immune cell function or maturation. Therefore, we further investigated the accumulation of these two major immune cell types in the plaques of *Lgmn* ^−/−^ *Apoe* ^−/−^ mice and *Lgmn* ^+/+^ *Apoe* ^−/−^ mice. Characterization of the immune cell composition in the plaques revealed a significant reduction in CD3^+^ T cells (Figure 1g,h). The relative number of macrophages in the plaques was slightly lower in HFD‐fed *Lgmn* ^−/−^ *Apoe* ^−/−^ mice after 12 weeks. However, no statistical difference was observed (Figure S2A,B). To investigate whether legumain plays a role in macrophage polarization in vivo, aortic root sections from *Lgmn* ^+/+^ *Apoe* ^−/−^ mice and *Lgmn* ^−/−^ *Apoe* ^−/−^ mice were immunostained for CD68 and arginase 1 (Arg‐1, M2 marker). Immunostaining analysis revealed decreased Arg‐1 expression in plaque CD68^+^ cells from *Lgmn* ^−/−^ *Apoe* ^−/−^ mice, suggesting that legumain deficiency in macrophages reduces M2 polarization. Altered macrophage polarization in the aortic root plaques of *Lgmn* ^−/−^ *Apoe* ^−/−^ mice cannot explain the reduced plaque burden (Figure S2A,B). These results suggest that legumain deficiency halts atherogenesis in *Apoe* ^−/−^ mice, which may be related to altered T‐cell immune responses.

### 2.2 Legumain deficiency reduces T cell accumulation in plaques

**FIGURE 2:**
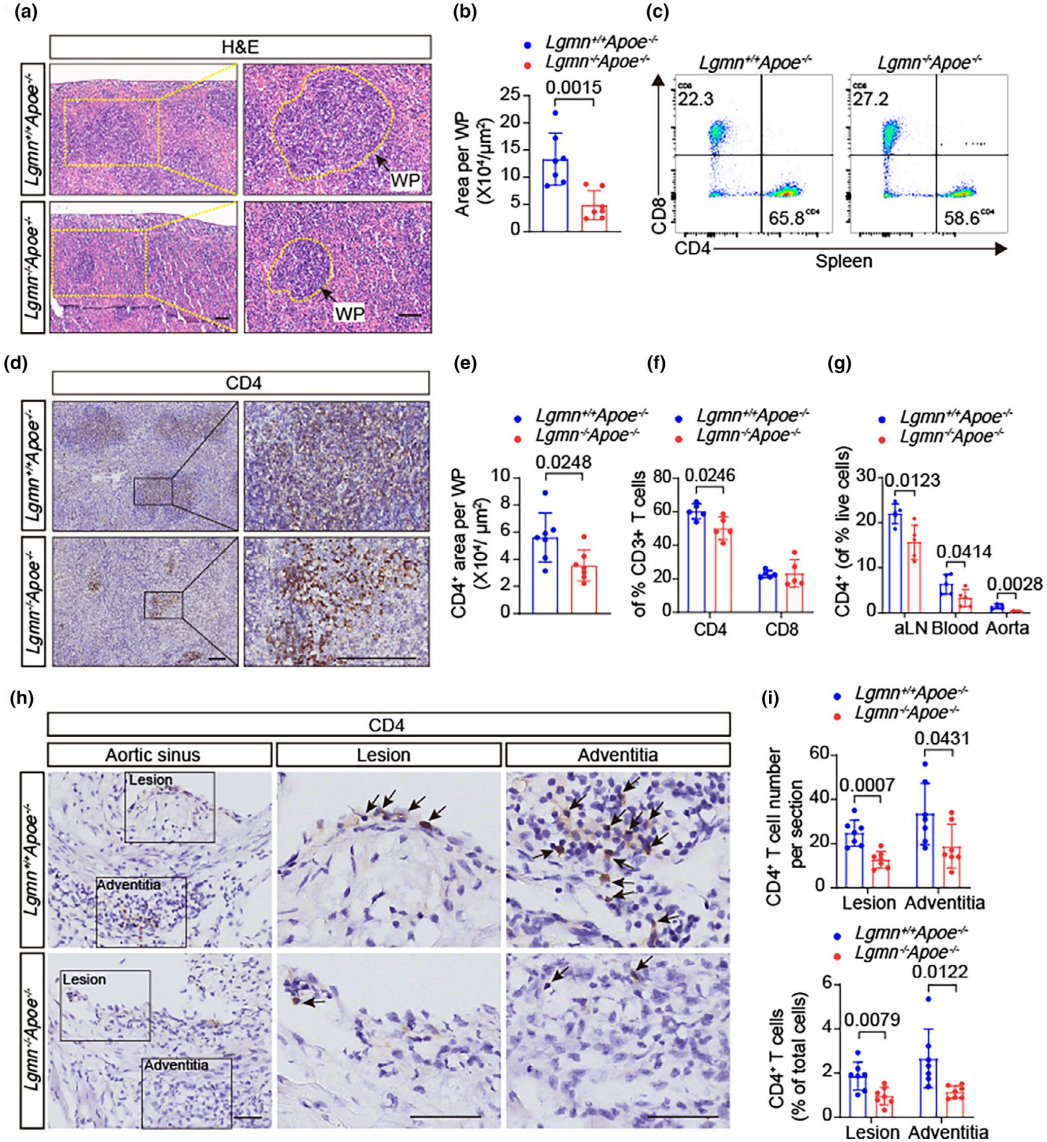
Legumain deficiency leads to loss of mature CD4^+^ T lymphocytes and reduces accumulation of CD4^+^ T cells in atherosclerotic plaques. (a, b) Representative images of hematoxylin and eosin (H&E)‐stained splenic white pulp (WP) from each group. Scale bar, 100 μm. Areas of white pulp in the spleen of each group were quantified (*n* = 7 mice per group). (c, f) Representative flow cytometry gating strategies for CD4^+^ T cells and CD8^+^ T cells. The proportions of CD4^+^ and CD8^+^ cells among splenic T cells from *Lgmn* ^+/+^ *Apoe* ^−/−^ mice and *Lgmn* ^−/−^ *Apoe* ^−/−^ mice were assessed by flow cytometry (*n* = 5 mice per group). (d, e) Representative immunohistochemistry images showing CD4^+^ cells in the spleens of *Lgmn* ^+/+^ *Apoe* ^−/−^ mice and *Lgmn* ^−/−^ *Apoe* ^−/−^ mice. Scale bar, 200 μm. The ratio of the CD4^+^ area to the WP area was quantified (*n* = 7 mice per group). (g) Relative distributions of CD4^+^ cells and CD8^+^ cells in the aorta‐draining lymph nodes (aLNs), blood, and aorta of *Lgmn* ^+/+^ *Apoe* ^−/−^ mice and *Lgmn* ^−/−^ *Apoe* ^−/−^ mice (*n* = 5 mice per group). (h, i) Representative immunohistochemical images showing CD4‐positive areas (indicated by arrows), and the number of CD4^+^ T cells in atherosclerotic lesions and the surrounding adventitia of the mice was quantified (*n* = 7 mice per group). Nuclei were stained with hematoxylin. Scale bar, 50 μm. H&E, hematoxylin and eosin; WP, white pulp. Data information: The exact *p* value is specified. The *p* value was determined by an unpaired two‐tailed Student's *t* test (b, e, f, g, and i).

To elucidate the role of legumain in T lymphocytes, we analyzed the histology of the spleen. H&E staining revealed that the area of the splenic white pulp was significantly reduced in *Lgmn* ^−/−^ *Apoe* ^−/−^ mice (Figure 2a,b). Considering the increased spleen weight in *Lgmn* ^−/−^ *Apoe* ^−/−^ mice, these results suggest that *Lgmn* ^−/−^ *Apoe* ^−/−^ mice have reduced lymphocyte density compared with *Lgmn* ^+/+^ *Apoe* ^−/−^ mice. We also used flow cytometry to detect the enrichment of T cells in the spleens of the mice and found that, in *Lgmn* ^−/−^ *Apoe* ^−/−^ mice, the percentage of CD3^+^ T cells among the spleen cells was reduced to 65.7% of the control group (Figure S3A,B). Consistently, the relative numbers of CD4^+^ cells and CD8^+^ T cells in the spleen were reduced (Figure S3C–F). Interestingly, when the percentages of CD4^+^ T cells and CD8^+^ T cells among the total CD3^+^ T cells were further analyzed, the relative decrease in CD4^+^ T cells was found to be more significant (0.83‐fold) (Figure 2c,f). Similar reductions in CD4 expression were observed at the mRNA level in the aortas of *Lgmn* ^−/−^ *Apoe* ^−/−^ mice (Figure S3G). The thymus is responsible for the development of CD4^+^ and CD8^+^ T cells. Interestingly, the number of single‐positive cells was similar in the thymuses of *Lgmn* ^−/−^ *Apoe* ^−/−^ mice and *Lgmn* ^+/+^ *Apoe* ^−/−^ mice (Figure S3H).

Because legumain deficiency strongly affects peripheral CD4^+^ T cell counts, we next focused on whether legumain deficiency affects the biology of CD4^+^ T cells in the periphery and aorta. Consistent with the results of the flow cytometry analysis, immunohistochemical staining revealed a dramatic decrease in the number of CD4^+^ T cells in the splenic white pulp of *Lgmn* ^−/−^ *Apoe* ^−/−^ mice (Figure 2d,e). CD4^+^ T cells were also decreased in the aorta‐draining lymph nodes, blood, and aorta of *Lgmn* ^−/−^ *Apoe* ^−/−^ mice, suggesting that legumain deficiency leads to a decrease in the number of peripheral mature CD4^+^ T cells (Figure 2g, Figure S3I–K). Moreover, CD4^+^ T cell accumulation in aortic plaques and surrounding adventitia was reduced by approximately 50% in *Lgmn* ^−/−^ *Apoe* ^−/−^ mice compared with *Lgmn* ^+/+^ *Apoe* ^−/−^ mice (Figure 2h,i). Overall, legumain deficiency reduces the density of CD4^+^ T cells in the periphery and in plaques, which may account for the suppression of atherogenesis.

### 2.3 Legumain deficiency inhibits TCR signaling in CD4 ^+^ T cells

**FIGURE 3:**
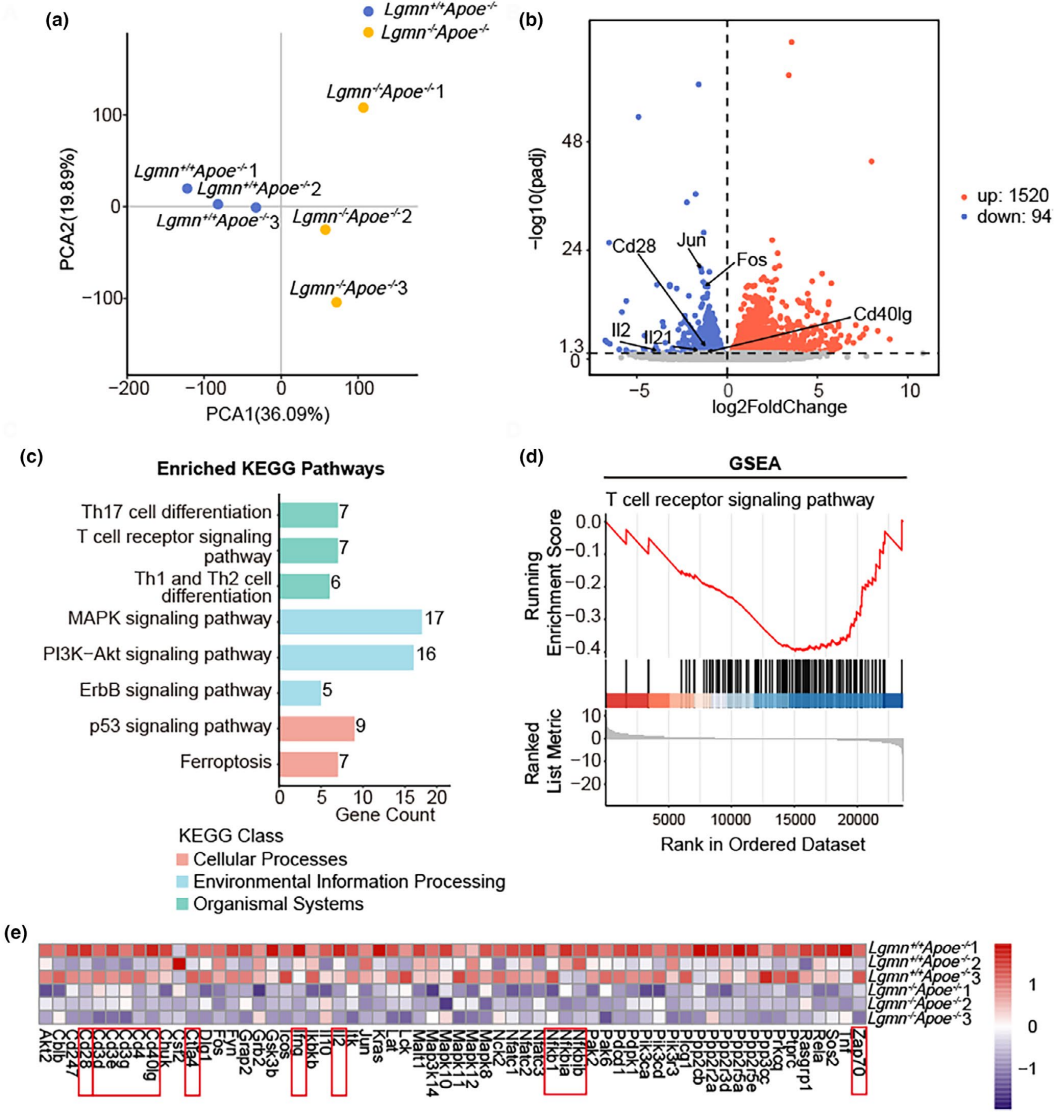
Legumain deficiency blocks the T cell receptor signaling (TCR) pathway in CD4^+^ T cells. (a) Principal component analysis (PCA) comparing splenic CD4^+^ T cells from *Lgmn* ^+/+^ *Apoe* ^−/−^ mice and *Lgmn* ^−/−^ *Apoe* ^−/−^ mice. (b) Volcano plot showing genes differentially expressed in splenic CD4^+^ T cells from *Lgmn* ^+/+^ *Apoe* ^−/−^ mice and *Lgmn* ^−/−^ *Apoe* ^−/−^ mice. (c) Kyoto Encyclopedia of Genes and Genomes (KEGG) pathway enrichment analysis of splenic CD4^+^ T cells from *Lgmn* ^+/+^ *Apoe* ^−/−^ mice and *Lgmn* ^−/−^ *Apoe* ^−/−^ mice. (d) Gene set enrichment analysis (GSEA) showing downregulation of the TCR signaling pathway. (e) Heatmap of genes significantly altered in the TCR signaling pathway (red = high, blue = low). PCA, principal component analysis.

To elucidate the mechanisms underlying the reduction in CD4^+^ T cells induced by legumain deficiency, we performed transcriptomic profiling of CD4^+^ T cells isolated from the spleens of *Lgmn* ^−/−^ *Apoe* ^−/−^ mice and *Lgmn* ^+/+^ *Apoe* ^−/−^ mice. The two groups presented distinct transcriptional profiles (Figure 3a). Differential expression analysis revealed a number of T cell activation‐ and differentiation‐related genes that were significantly downregulated in legumain‐deficient T cells (Figure 3b). All differentially expressed genes (DEGs) between *Lgmn* ^−/−^ *Apoe* ^−/−^ and *Lgmn* ^+/+^ *Apoe* ^−/−^ mice were annotated by Kyoto Encyclopedia of Genes and Genomes (KEGG) pathway enrichment analysis, which revealed that the DEGs were highly enriched in pathways related to T cell activation, such as the TCR signaling pathway, downstream MAPK signaling pathway, and PI3K‐Akt signaling pathway. In addition, some DEGs were enriched in the regulation of T cell differentiation (Figure 3c). To further determine whether DEGs were differentially regulated in legumain‐deficient and control T cells, gene expression data were functionally interpreted and characterized using gene set enrichment analysis (GSEA). GSEA revealed that the expression of TCR signaling pathway genes was downregulated in T cells from *Lgmn* ^−/−^ *Apoe* ^−/−^ mice compared to T cells from *Lgmn* ^+/+^ *Apoe* ^−/−^ mice (Figure 3d). By reviewing the detailed information of the genes within this term, we identified a number of DEGs associated with the TCR signaling pathway, such as Cd28 and Cd40lg, which encode co‐stimulatory molecules; Zap70, which encodes a key molecule in TCR signaling; and Ifng, which encodes interferon‐γ (IFN‐γ). The heatmap illustrates the expression patterns of 62 DEGs within the TCR signaling pathway in the two groups (Figure 3e). Taken together, these results suggest that legumain deficiency induces the downregulation of genes related to the TCR signaling pathway in the transcriptome of CD4^+^ T cells.

### 2.4 Legumain deficiency suppresses survival and promotes apoptosis of CD4^+^ T cells

**FIGURE 4:**
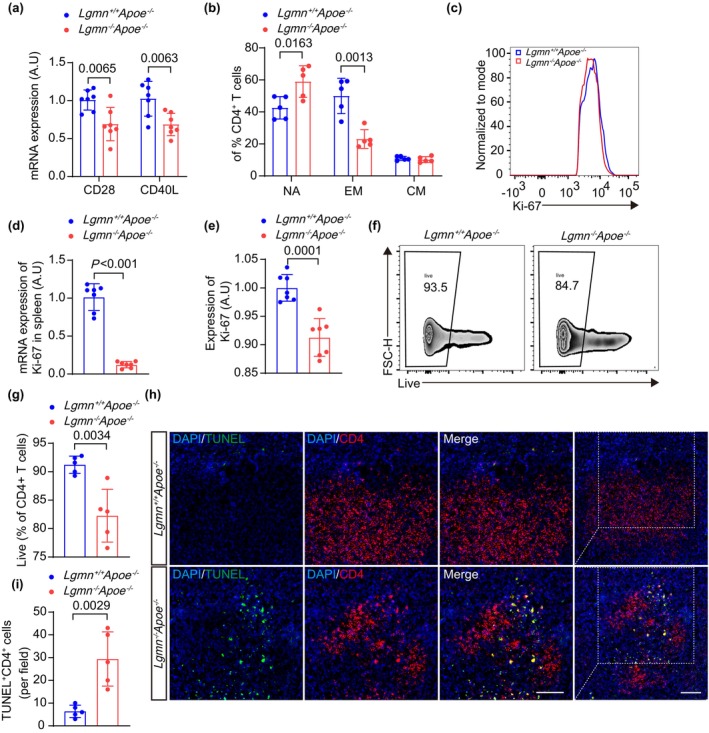
Deletion of legumain reduces survival and increases apoptosis of CD4+ T cells (a) Quantification of co‐stimulatory molecule expression in CD4^+^ T cells from each group. The data were obtained by quantitative real‐time PCR (*n* = 7 mice per group). (b) The percentages of CD44^−^CD62L^+^ naive (NA), CD44^+^CD62L^−^ effector memory (EM), and CD44^+^CD62L^+^ central memory (CM) T cells in gated splenic CD4^+^ T cells from *Lgmn* ^+/+^ *Apoe* ^−/−^ mice and *Lgmn* ^−/−^ *Apoe* ^−/−^ mice were quantified (*n* = 5 mice per group). (c–e) Representative flow cytometry histograms for Ki‐67. The expression of Ki‐67 in CD4^+^ T cells in each group was assessed by quantitative real‐time PCR and flow cytometry analysis (*n* = 7 mice per group). (f, g) Representative flow cytometry gating strategies for Annexin V staining. The percentage of live CD4^+^ T cells in each group was assessed directly at harvest (*n* = 5 mice per group). (h, i) TdT‐mediated dUTP nick‐end labeling (TUNEL) apoptosis assay. Double‐labeling immunofluorescence of TUNEL (green) and CD4 (red) in spleen sections from *Lgmn* ^+/+^ *Apoe* ^−/−^ mice and *Lgmn* ^−/−^ *Apoe* ^−/−^ mice. The number of TUNEL^+^ CD4^+^ T cells in each field was quantified (*n* = 5 mice per group). CM, central memory; EM, effector memory; NA, naive T cells; TUNEL, TdT‐mediated dUTP nick‐end labeling. Data information: The exact *p* value is specified. The *p* value was determined by an unpaired two‐tailed Student's *t* test (a, b, d, e, g, and i).

Since TCR signaling was downregulated in legumain‐deficient T cells according to transcriptome sequencing, we further investigated the regulation of TCR signaling by legumain deficiency in mouse CD4^+^ T cells. We first validated the co‐stimulatory molecules required for the initial stimulation and activation of T cells. Reduced CD28 and CD40L mRNA expression in CD4^+^ T cells from the spleens of *Lgmn* ^−/−^ *Apoe* ^−/−^ mice was confirmed by quantitative real‐time PCR (qRT–PCR) (Figure 4a). Moreover, by analyzing the expression of CD44 and CD62L in CD4^+^ T cells, we evaluated the effect of legumain deficiency on T cell activation. Detailed phenotyping revealed that legumain deficiency reduced the percentage of effector memory (EM; CD4^+^CD44^+^CD62L^−^) T cells among CD4^+^ T cells in *Apoe* ^−/−^ mice (0.44‐fold) (Figure 4b). Accordingly, an increase in naive (NA; CD4^+^CD44^−^CD62L^+^) T cells among CD4^+^ T cells was observed in the spleens of *Lgmn* ^−/−^ *Apoe* ^−/−^ mice (Figure 4b). The reduction in CD4^+^ T cell abundance in *Lgmn* ^−/−^ *Apoe* ^−/−^ mice may be due to impaired TCR signaling leading to reduced proliferation. This finding was supported by the significant reduction in the protein and mRNA expression levels of Ki‐67 (a marker of cellular proliferative activity) in CD4^+^ T cells (Figure 4c–e). Assessment of apoptosis by flow cytometry revealed a dramatic increase in the percentage of apoptotic CD4^+^ T cells (annexin V^+^) in the spleens of *Lgmn* ^−/−^ *Apoe* ^−/−^ mice (0.90‐fold) (Figure 4f,g). Furthermore, an increase in the number of apoptotic cells was detected in atherosclerotic plaques and in the spleen by an in situ TdT‐mediated dUTP nick end labeling (TUNEL) assay in *Lgmn* ^−/−^ *Apoe* ^−/−^ mice (Figure 4h,i, Figure S4A,B). These results suggest that the deletion of legumain attenuates T cell proliferation and increases apoptosis, potentially serving as a critical mechanism underlying the decline in CD4^+^ T cells.

### 2.5 Legumain deficiency modulates the CD4 ^+^ T cell phenotype in *Apoe* ^*−/−*^ mice

**FIGURE 5:**
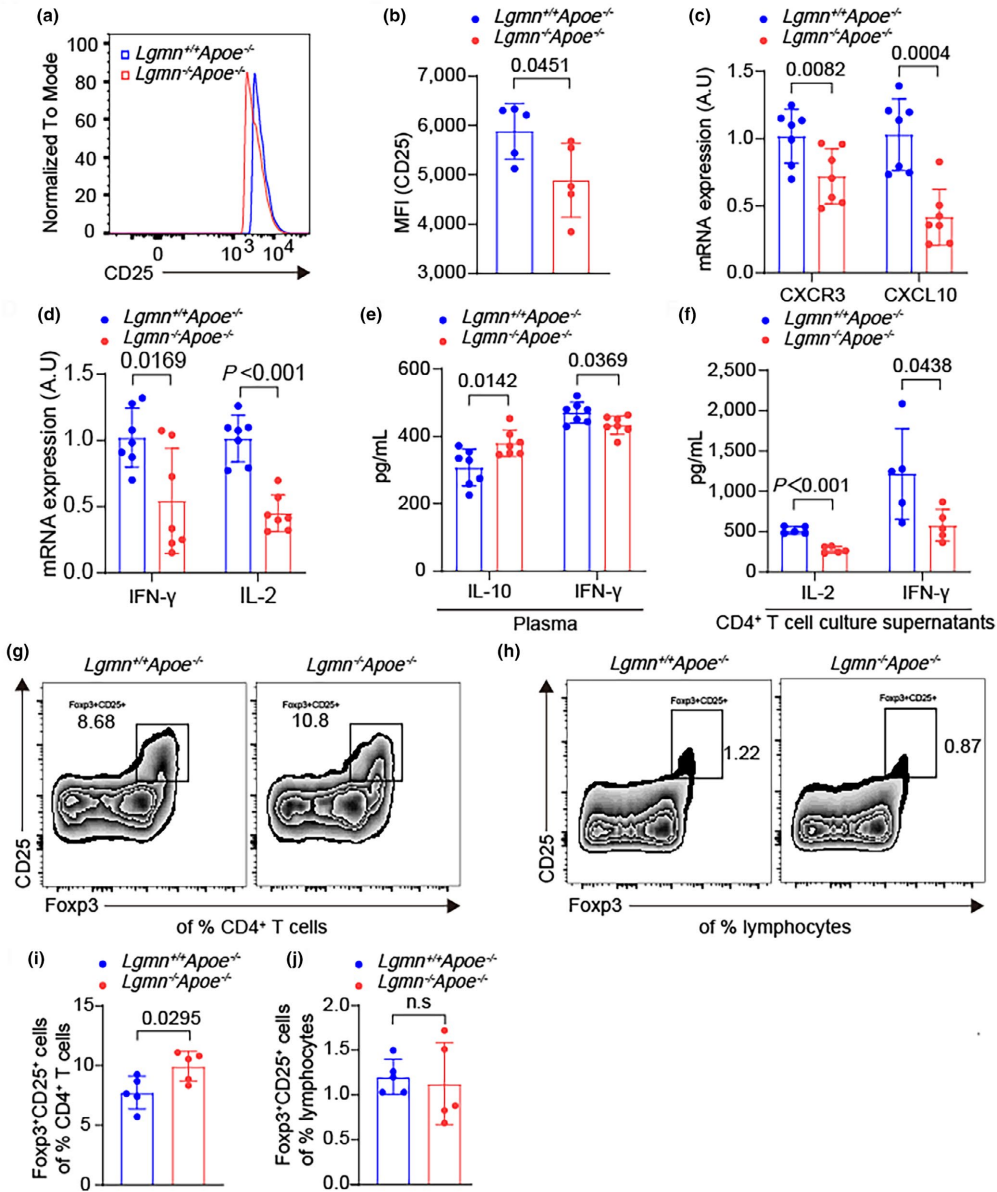
Legumain deficiency reduces CD4^+^ T‐cell activation and impairs T‐cell function. (a, b) Flow cytometry analysis of CD25 in splenic CD4^+^ T cells from *Lgmn* ^+/+^ *Apoe* ^*−/−*^ mice and *Lgmn* ^−/−^ *Apoe* ^*−/−*^ mice together with representative flow cytometry histograms for CD25 (*n* = 5 mice per group). (c, d) Expression of activation markers, including C‐X‐C motif chemokine 3 (CXCR3) and its ligand C‐X‐C motif chemokine ligand 10 (CXCL10), as well as the cytokines interferon (IFN)‐γ and interleukin (IL)‐2 (*n* = 7 mice per group), in CD4+ T cells stimulated for 24 h was analyzed by quantitative real‐time PCR (24 h). (e, f) Levels of interleukin (IL)‐10, IFN (interferon)‐γ, and (IL)‐2 in the plasma (*n* = 7 mice per group) and culture supernatants (*n* = 5 mice per group) of activated spleen CD4^+^ T cells in each group were analyzed via ELISA. (g–j) Representative flow cytometry analysis and quantification of the percentage of CD4^+^CD25^+^Foxp3^+^ Tregs among splenic CD4^+^ T cells and lymphocytes from *Lgmn* ^+/+^ *Apoe* ^−/−^ mice and *Lgmn* ^−/−^ *Apoe* ^−/−^ mice (*n* = 5 mice per group). Data information: The exact *p* value is specified. The *p* value was determined by an unpaired two‐tailed Student's *t* test (b–f, i, and j).

Previous studies have shown that activation of the TCR signaling pathway promotes T‐cell survival and proliferation (Nitz et al., 2022). Consistent with the impaired T cell activation observed in *Lgmn* ^−/−^ *Apoe* ^−/−^ mice, a decrease in the expression of CD25 (an indicator of T cell activation) on *Lgmn* ^−/−^ *Apoe* ^−/−^CD4^+^ T cells was also observed (Figure 5a,b). We also examined the transcript levels of other activation markers of CD4^+^ T cells in the spleen, including C‐X‐C motif chemokine receptor 3 (CXCR3) and its ligand C‐X‐C motif chemokine ligand 10 (CXCL10), as well as the cytokines IFN‐γ and IL‐2. The mRNA levels of activation markers were lower in splenic CD4^+^ T cells from *Lgmn* ^−/−^ *Apoe* ^−/−^ mice than in those from control mice (Figure 5c,d). Moreover, we detected decreased IFN‐γ and increased IL‐10 levels in the plasma of *Lgmn* ^−/−^ *Apoe* ^−/−^ mice fed a HFD for 12 weeks, as well as decreased IFN‐γ and IL‐2 secretion in the supernatants of CD3/IL‐2‐activated splenic CD4^+^ T cells isolated from *Lgmn* ^−/−^ *Apoe* ^−/−^ mice (Figure 5e,f). Since CD25 is also a phenotypic marker of regulatory T (Treg) cells, we further investigated whether the reduction in CD25 expression observed in *Lgmn* ^−/−^CD4^+^ T cells might be associated with the number of Treg cells. Legumain deficiency increased the proportion of Foxp3^+^CD25^+^ Treg cells among total CD4^+^ T cells but did not affect the proportion of Treg cells among splenic lymphocytes (Figure 5g–j). Overall, legumain deficiency results in reduced T‐cell migration to the aorta and the production of fewer proinflammatory cytokines, all of which may contribute to a reduction in T‐cell numbers and atherosclerotic plaques.

### 2.6 Legumain deficiency downregulates B‐cell lymphoma 2 (Bcl‐2) expression and increases oxidative stress

**FIGURE 6:**
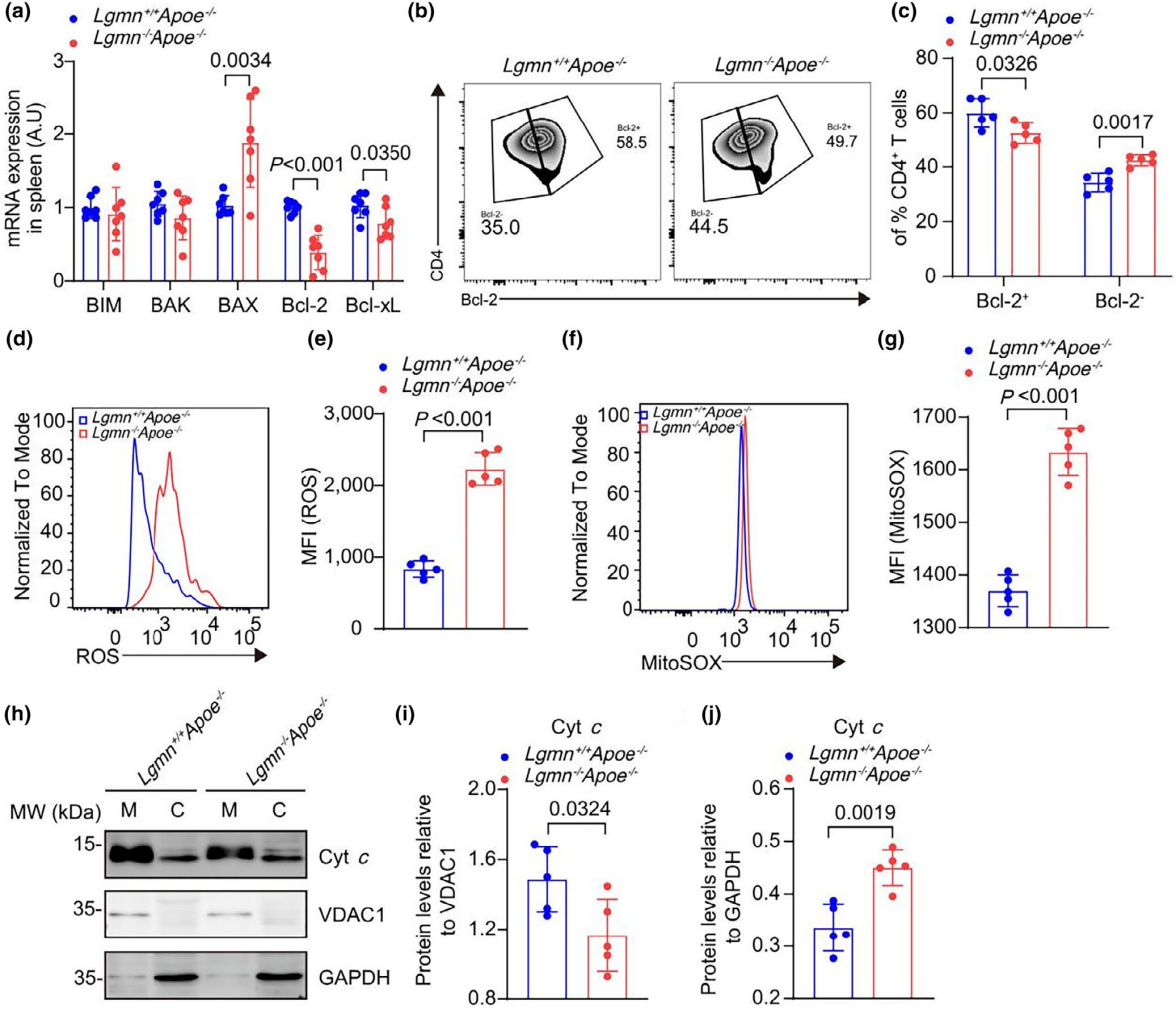
Legumain deficiency decreases Bcl‐2 expression and increases oxidative stress in CD4^+^ T cells. (a) The mRNA expression of the indicated pro‐ and anti‐apoptotic proteins on activated CD4^+^ T cells was quantified (*n* = 7 mice per group). (b, c) Representative flow cytometry analysis and quantification of the percentages of CD4^+^Bcl‐2^−^ T cells and CD4^+^Bcl‐2^+^ T cells among splenic CD4^+^ T cells (*n* = 5 mice per group). (d, e) Flow cytometry analysis of the levels of ROS in stimulated splenic CD4^+^ T cells from *Lgmn* ^+/+^ *Apoe* ^−/−^ mice and *Lgmn* ^−/−^ *Apoe* ^−/−^ mice together with representative flow cytometry histograms for reactive oxygen species (ROS) (*n* = 5 mice per group). (f, g) Flow cytometry analysis of the levels of mitoROS via mitoSOX in stimulated splenic CD4^+^ T cells from *Lgmn* ^+/+^ *Apoe* ^−/−^ mice and *Lgmn* ^−/−^ *Apoe* ^−/−^ mice together with representative flow cytometry histograms for mitoROS (*n* = 5 mice per group). (h) Representative Western blot showing the expression of cytochrome c (Cyt c) in the mitochondrial fraction or cytosolic fraction of splenic CD4^+^ T cells from *Lgmn* ^+/+^ *Apoe* ^−/−^ mice and *Lgmn* ^−/−^ *Apoe* ^−/−^ mice. (i, j) The relative levels of mitochondrial or cytosolic Cyt c in splenic CD4^+^ T cells from *Lgmn* ^+/+^ *Apoe* ^−/−^ mice and *Lgmn* ^−/−^ *Apoe* ^−/−^ mice. Voltage‐Dependent Anion Channel 1 (VDAC1) was used as a loading control for the mitochondrial fraction. GAPDH was used as a loading control for the cytosolic fraction (*n* = 5 mice per group). BAK, Bcl‐2 homologous antagonist/killer; BAX, Bcl‐2‐associated X; BIM, Bcl‐2 interacting mediator of cell death; Cyt c, cytochrome c; ROS, reactive oxygen species; VDAC1, Voltage‐Dependent Anion Channel 1. Data information: The exact *p* value is specified. The *p* value was determined by an unpaired two‐tailed Student's *t* test (a, c, e, g, i, and j).

Downstream of T cell activation, Bcl‐2 family members maintain long‐term T cell survival and promote memory cell differentiation (Song et al., 2008; Tang et al., 2019). Significant reductions in the mRNA levels of Bcl‐2 (61%) and B‐cell lymphoma extra‐large (Bcl‐XL) (24%) were detected in *Lgmn* ^−/−^CD4^+^ T cells after activation in vitro, whereas the levels of the Bcl‐2 interacting mediator of cell death (BIM) and the Bcl‐2 homologous antagonist/killer (BAK) were comparable to those in control cells. Increased transcription levels of the pro‐apoptotic protein Bcl‐2‐associated X (BAX) were also observed (1.83‐fold) (Figure 6a). Next, we measured the expression of the anti‐apoptotic protein Bcl‐2, a key effector of T‐cell activation. In response to CD3/IL‐2 stimulation, legumain deficiency reduced the proportion of anti‐apoptotic Bcl‐2^+^CD4^+^ T cells by approximately 12% and increased the proportion of pro‐apoptotic Bcl‐2^*−*^CD4^+^ T cells by approximately 1.24‐fold (Figure 6b,c). Upon activation of the Bcl‐2 pathway, mitochondrial reactive oxygen species (ROS) induce the release of cytochrome c from the mitochondria, leading to caspase activation (Chen & Pervaiz, 2007; Chong et al., 2014). Legumain deficiency increased ROS levels in CD4^+^ T cells (0.37‐fold), suggesting increased oxidative stress in *Lgmn* ^−/−^ CD4^+^ T cells (Figure 6d,e). In support of these findings, we detected increased levels of mitoROS and cytosolic cytochrome c in CD4^+^ T cells from *Lgmn* ^−/−^ *Apoe* ^−/−^ mice (Figure 6f–j). Taken together, these findings show that legumain deficiency reduces Bcl‐2 expression and increases cell damage. This may contribute to the decrease in peripheral CD4^+^ T cells.

## 3 DISCUSSION

In the present study, we investigated the expression of legumain in T cells within aortic atherosclerotic plaques. Legumain deficiency attenuated the plaque burden in *Apoe* ^−/−^ mice, which was associated with impaired maturation of peripheral CD4^+^ T cells and reduced T cell infiltration in the arterial wall. Mechanistically, legumain deficiency in CD4^+^ T cells inhibited T cell activation by downregulating the expression of co‐stimulatory molecules in the TCR pathway and the secretion of IL‐2. It also reduced the survival and proliferation of proinflammatory CD4^+^ memory T cells and downregulated the expression of chemokines on CD4^+^ T cells. Moreover, downregulation of Bcl‐2 by legumain deficiency via the TCR signaling pathway promoted increased apoptosis and ROS levels, thereby impairing cell survival. In conclusion, the present study demonstrated that legumain affects the progression of atherosclerosis by modulating CD4^+^ T cell biology, which is a novel immunological molecular mechanism of legumain in the pathogenesis of atherosclerosis.

Antigen‐presenting cells (APCs) expressing MHC class II molecules form the basis of the adaptive immune response in atherosclerosis (Hansson & Hermansson, 2011; Stemme et al., 1995). APCs within atherosclerotic plaques and adjacent adventitia capture plaque‐derived antigens and migrate to lymph nodes, where they present these antigens to naive T cells (Llodrá et al., 2003). In a mouse model of atherosclerosis, many CD4^+^ T cells in lymph nodes and atherosclerotic plaques exhibit a memory T cell phenotype, characterized by the expression of surface markers such as CD44. Changes in T‐cell immune activity reflect T‐cell immune dysfunction in atherosclerosis (Doran, 2022). These imbalances promote inflammation and alter macrophage polarization, smooth muscle cell migration, and endothelial dysfunction (Bazioti et al., 2023; Orecchioni et al., 2019; Zhang, Tang, et al., 2015). In this study, we found that legumain plays a role in activating and surviving CD4^+^ T cells and thus explored the correlation between legumain levels and altered atherosclerotic plaque size.

Legumain is a cysteine protease that contributes to the development of several neurodegenerative diseases by cleaving specific substrates, impairing physiological function, or producing pathogenic fragment proteins (Zhang et al., 2014, 2017; Zhang, Song, et al., 2015). It also plays a critical role in tumor progression, cardiac remodeling, and antigen processing (Jia et al., 2022; Pan et al., 2022; Zhang et al., 2023). Legumain is tightly regulated at the transcriptional levels. CEBPB CCAAT enhancer binding protein β (C/EBP β), an inflammatory cytokine‐activated transcription factor, is the major transcriptional factor that regulates legumain expression. With plaque progression, increased inflammation and lipid deposition promote C/EBP β activation and legumain expression (Cloutier et al., 2009; Liu et al., 2022). The increased spleen weight caused by legumain deficiency may be primarily due to increased haemophagocytosis as a result of legumain‐associated erythrocyte membrane defects (Chan et al., 2009). Moreover, intracellular substrate accumulation and increased lysosomal protein expression in the spleen may exacerbate splenomegaly in legumain‐deficient mice (Martínez‐Fábregas et al., 2018). Given the ability of legumain to promote tissue repair after myocardial infarction and the potential splenomegaly and erythropoiesis that may result from legumain downregulation (Jia et al., 2022), low levels of legume protease expression may be beneficial and should be further investigated. Previous literature suggests that legumain inhibitors partially block its activity (Zhang et al., 2017), providing an important clue for further research. Cell‐ or tissue‐specific legumain downregulation would be a better choice for clinical applications.

Two T cell activation signals are essential for the activation of naive T cells: the first is specific and is required for the recognition of MHC‐presenting antigens by TCR binding. The second is non‐specific and is generated by the binding of co‐stimulatory ligands expressed by APCs to receptors on T cells (Song et al., 2008). Without co‐stimulatory signals, whether from T cell receptors or ligands on APCs, T cells can undergo apoptosis or become anergic (Frauwirth & Thompson, 2002). CD28 plays an important role in T cell activation, differentiation, tolerance, and memory. Lack of CD28 co‐stimulatory signaling leads to the anergy of stimulated T cells (Ford et al., 2008). However, interactions between other co‐stimulatory molecules, such as the CD40/CD40 ligand (CD40L), are essential for the full activation of CD28‐deficient T cells (Ewing et al., 2013; Girvin et al., 2002). Our data showed that interfering with both CD28‐B7 and CD40‐CD40L interactions inhibits T cell activation and proliferation in T cells while allowing cell cycle‐dependent apoptosis to occur. This effect may be independent of the increase in IL‐10 in *Lgmn* ^−/−^ *Apoe* ^−/−^ mice, as the inhibition of co‐stimulatory signaling by IL‐10 is achieved by the down‐regulation of co‐stimulatory ligands on the surface of the APC (Shevach, 2009).

In conclusion, our study suggests that legumain plays a key role in regulating CD4^+^ T‐cell function and the progression of atherosclerosis. Legumain deficiency impairs the TCR signaling pathway and inhibits CD4^+^ T cell activation, survival, and proliferation, leading to a reduction in T cell accumulation in plaque lesions. These findings are clinically relevant because legumain expression is increased in plaques from patients with carotid atherosclerosis and may lead to plaque destabilisation. Therefore, targeting legumain in specific cells or tissues may be a potential therapeutic strategy to control the progression of atherosclerosis.

## 4 METHODS

### 4.1 Mice

*Lgmn* ^−/−^ (asparagine endopeptidase knockout) mice on a mixed 129/Ola and C57BL/6 background were generated as previously described (Shirahama‐Noda et al., 2003). Apolipoprotein E knockout (*Apoe* ^−/−^) mice (Vital River Animal Center) were crossed with *Lgmn* ^−/−^ mice to generate *Lgmn* ^−/−^ *Apoe* ^−/−^ mice. Male *Lgmn* ^−/−^ *Apoe* ^−/−^ mice and their littermate controls (*Lgmn* ^+/+^ *Apoe* ^−/−^) were subjected to a 12‐week HFD (#D12079B; Special Diet Services Ltd., Essex, UK) to induce atherosclerotic plaques. We examined male mice because the size of atherosclerotic plaques may be influenced by sex hormones (Arnold et al., 2017). *Lgmn* ^−/−^ *Apoe* ^−/−^ mice and control littermates were housed in SPF‐grade clean animal rooms with free access to food and water. The mice were euthanized by inhalation of carbon dioxide at increasing concentrations. Experimental procedures were approved by the Institutional Animal Care and Use Committee (IACUC) of Tongji Medical College, Huazhong University of Science and Technology, with an IACUC issue number of 3502. All animal experiments in this study were performed in accordance with the United States National Institutes of Health Guide for the Care and Use of Laboratory Animals.

### 4.2 Histological analysis

The mice were euthanized via CO_2_ gas inhalation after 12 weeks of HFD feeding. After the intracardial perfusion with PBS, the mouse hearts were excised and stored in 4% paraformaldehyde solution for at least 24 h until use. The aortic root was sectioned parallel to the lumen into 6.0 μm thick slices and placed on glass slides. The tissue sections were subjected to hematoxylin and eosin (H&E) staining, immunofluorescence, and immunohistochemistry. Images were captured with an Olympus optical microscope (Olympus, Japan). We took the appearance of the 3 aortic valve leaflets as the starting point and selected the middle 3 of the consecutive posterior sections for analysis. Statistical analysis was performed by experimenters who were unaware of the groupings of the mice. Histological analysis of atherosclerotic lesions was performed using ImageJ software.

### 4.3 Serum lipid analyses

After an overnight fast, 100 μL of mouse blood was collected via the mandibular vein, and the serum was stored at −80°C until use. Lipid analysis was performed using a lipid kit from Nanjing Jianjian Bioengineering Institute (Total Cholesterol Assay Kit, A111‐1‐1; Triglyceride Assay Kit, A110‐1‐1; High‐density lipoprotein cholesterol Assay Kit, A112‐1‐1; and Low‐density lipoprotein cholesterol Assay Kit, A113‐1‐1). For the determination of total cholesterol (TC) and triglyceride (TG) levels in the serum, the serum was incubated in reaction buffer at 37°C for 10 min, after which the absorbance was read at 510 nm. For the determination of high‐density lipoprotein cholesterol (HDL‐c) and low‐density lipoprotein cholesterol (LDL‐c) in the serum, the serum was incubated twice in the corresponding reaction buffer at 37°C for 5 min, and the absorbance was read at 546 nm.

### 4.4 Isolation of cells

After the mice were euthanized, whole blood was collected, and white blood cells were isolated in centrifuge tubes. The residual erythrocytes were removed from the erythrocyte lysate (Solarbio, R1010) and washed three times with staining buffer. The spleen, thymus, and aorta‐draining lymph nodes were excised, ground in a cell strainer (352350, Corning, 70 μm), and washed with staining buffer (BD Pharmingen, 554656). To isolate immune cells in the aorta, the aorta was excised after PBS perfusion, cut into small pieces, and then digested with type I collagenase, elastase, and DNase at 37°C for 30 min. The mixture was then filtered through a cell strainer (70 μm).

### 4.5 Flow cytometry

The Fixable Viability Stain 510 Reagent (BD Pharmingen, 564406) was included in the antibody panels as needed. Fc‐blocked anti‐CD16/32 (details in Table S2) was added directly to the cells before staining. The cells were stained with diluted antibodies against CD3, CD4, CD8, CD44, CD62L, and CD25 (details in Table S2) for 20 min at 4°C in staining buffer (BD Pharmingen, 554656). To examine the expression of the intracellular proteins Bcl‐2, Ki‐67, and Foxp3 (details in Table S2), the cells were pretreated with a Transcription Factor Buffer Set (BD Pharmingen, 562574). After washing, the cells were subjected to flow cytometric analysis (BD LSRFortessa™ X‐20 Special Order Product) to determine the expression of the proteins. Apoptosis staining was performed via an Annexin V Apoptosis Detection Kit (Elabscience, E‐CK‐A212) according to the manufacturer's instructions. The data were analyzed via FlowJo software (V10.8.1).

### 4.6 Cell activation and functional analysis

CD4^+^ T cells were isolated from the spleens of 12‐week HFD–induced *Lgmn* ^−/−^ *Apoe* ^−/−^ mice and *Lgmn* ^+/+^ *Apoe* ^−/−^ mice using antibody‐conjugated magnetic beads according to the manufacturer's instructions (Dynabeads™ Untouched™ Mouse CD4 Cell Kit, Thermo Fisher Scientific, 11416D). To induce proliferation, 200 μL of a CD4^+^ T cell suspension containing IL‐2 (10 ng/mL, MCE, HY‐P7077) was seeded onto plates supplemented with a CD3 antibody (5 μg/mL, Biolegend, 100340).

### 4.7 Quantification of ROS

To quantify ROS, the cells were treated with a fluorescent dye called 2,7‐dichlorofuorescin diacetate (DCFH‐DA) at a concentration of 5 μM. DCFH‐DA was obtained from Nanjing Jiancheng Bioengineering Institute (ROS Assay Kit, E004‐1‐1). After being incubated for 20 min, the cells were rinsed with PBS. The stained cells were then collected in PBS, and their fluorescence intensities were measured via a BD LSRFortessa™ X‐20 instrument. Finally, FlowJo software (V10.8.1) was used to analyze the acquired data.

### 4.8 Mitochondrial ROS assay

Splenic CD4^+^ T cells from *Lgmn* ^+/+^ *Apoe* ^−/−^ mice and *Lgmn* ^−/−^ *Apoe* ^−/−^ mice were harvested and stained with 2 μM MitoSOX Red (MCE, HY‐D1055) for 30 min at 37°C. The fluorescence intensities were measured via flow cytometry using a BD LSRFortessa™ X‐20 instrument. The data were analyzed via FlowJo software (V10.8.1).

### 4.9 Isolation of subcellular fractions

Cytosolic and mitochondrial fractions were isolated via a mitochondria extraction kit (Solarbio, Cat: SM0020) according to the manufacturer's instructions.

### 4.10 Immunofluorescence and immunohistochemical staining

Aortic root sections were retrieved at −20°C, rewarmed for 30 min, and then permeabilized with 0.3% Triton‐X. The tissue was blocked with 10% donkey serum. Primary antibodies against different species were coincubated with tissue sections at 4°C overnight. For IF staining, the samples were incubated with a fluorescently labeled secondary antibody the following day. 4′,6‐Diamidino‐2‐phenylindole (DAPI; Beyotime, C1005) was used to label the cell nuclei. Immunofluorescence images were captured using a Nikon confocal microscope to visualize signals in the sections. For immunohistochemical staining, the signal was generated according to the manufacturer's instructions for the High‐Efficiency IHC Detection System Kit (Absin, abs957). The antibody target antigens and working concentrations are provided in Table S2. ImageJ software was used for data analysis.

### 4.11 TUNEL assays

For TUNEL assays (Elabscience, E‐CK‐A420), sections were retrieved at −20°C, rewarmed for 30 min, permeabilized with 0.3% Triton‐X, and blocked with 10% donkey serum. Equilibration buffer was added to the samples for 5 min at RT before they were incubated with 35 μL of equilibration buffer containing 10 μL of labeling solution and 5 μL of TdT enzyme for 2 h at 37°C. The reaction was stopped by washing three times with PBS. DAPI was used to label the cell nuclei. ImageJ software was used for data analysis.

### 4.12 Quantitative real‐time PCR

TRIzol reagent was used to extract total RNA from CD4^+^ T cells and tissues (Invitrogen, 15596026). Total RNA (1000 ng) was subjected to reverse transcription into complementary DNA (cDNA) using the HiScript IV RT SuperMix Kit (Vazyme, R423‐01). ChamQ SYBR qPCR Master Mix Kit (Vazyme, Q311‐02) was utilized for quantitative real‐time PCR (qRT‐PCR), which was performed in triplicate and repeated at least three times independently. Sequences of gene‐specific primers were obtained from PrimerBank and synthesized by Sangon Biotech (details in Table S3). GAPDH was used as a control, and the relative fold change in expression was calculated using the comparative CT method (Livak & Schmittgen, 2001).

### 4.13 RNA sequencing

Total RNA was extracted using TRIzol (Invitrogen, 15596026) according to the supplied instructions. PCR amplification was performed on cDNA fragments obtained from reverse transcription, and the products were purified using Ampure XP DNA. Quality control was performed on an Agilent Technologies 2100 Bioanalyzer. The final library was generated by subjecting the double‐stranded PCR products to heating, denaturation, and cyclization facilitated by spliced oligonucleotides.

### 4.14 Statistical analysis

The data are expressed as the mean ± SEM. The normality of the data was determined by the Shapiro–Wilk normality test. For normally distributed data, unpaired two‐tailed Student's *t* test (for two‐group comparisons) was employed. Non‐parametric data were analyzed using the Mann–Whitney *U* test (for two groups). The determination of the quantity of experimental animals and the number of experiments per group was based on our expertise and previous research. A *p* value <0.05 was considered statistically significant, with detailed information provided in the figures. All the statistical analyses were performed with GraphPad Prism (version 8.0).

## AUTHOR CONTRIBUTIONS

XX and FZ performed the experiments and wrote the manuscript. LN, XG, and MQ participated in data collection. JC and DJ helped with mouse crosses and genotyping. ZZ contributed to the conception and revision of the manuscript. LM conceived the article, revised the manuscript, and approved the final manuscript. With FZ's permission, XX was assigned the first position in the authorship order because XX contributed to the original idea of this manuscript.

## FUNDING INFORMATION

This work was supported by the National Natural Science Foundation of China (no. 82171325 to Ling Mao).

## CONFLICT OF INTEREST STATEMENT

The authors declare no conflicts of interest.

## Supporting information


Appendix S1.

## Data Availability

The data that support the findings of this study are available from the corresponding author upon reasonable request.
