# Supplementary material for: Legumain deficiency halts atherogenesis by modulating T cell receptor signaling
Source: Aging Cell. 2024 Oct 29;24(2):e14391. doi: 10.1111/acel.14391 (PMC11822642; doi:10.1111/acel.14391)
Supplement: Supplementary file 1 — Appendix S1. [file ACEL-24-e14391-s001.zip › Appendix S1/Supplementary File.docx]

**Supplementary File**

**Legumain Deficiency Halts Atherogenesis by Modulating T Cell Receptor Signaling**

Xuying Xiang,^1,#^ Feng Zhang,^1,#^ Lei Nie,^1^ Xiaoqing Guo,^1^ Mengting Qin,^1^ Jiaojiao Chen,^1^ Dailiang Jiang,^1^ Zhentao Zhang,^2^ Ling Mao^1*^

^1^Department of Neurology, Union Hospital, Tongji Medical College, Huazhong University of Science and Technology, Wuhan, 430022, China.

^2^Department of Neurology, Renmin Hospital of Wuhan University, Wuhan， 430060, China.

^#^These authors contributed equally: Xuying Xiang, Feng Zhang.

*Corresponding author:

Ling Mao, M.D., Ph.D.

Department of Neurology, Union Hospital, Tongji Medical College, Huazhong University of Science and Technology

1277 Jiefang Avenue, Wuhan, 430022, China

Tel: +86-15172314891

E-mail: maoling@mail.hust.edu.cn

**Contents:**

Figures S1-S4

Table S1-S3

**
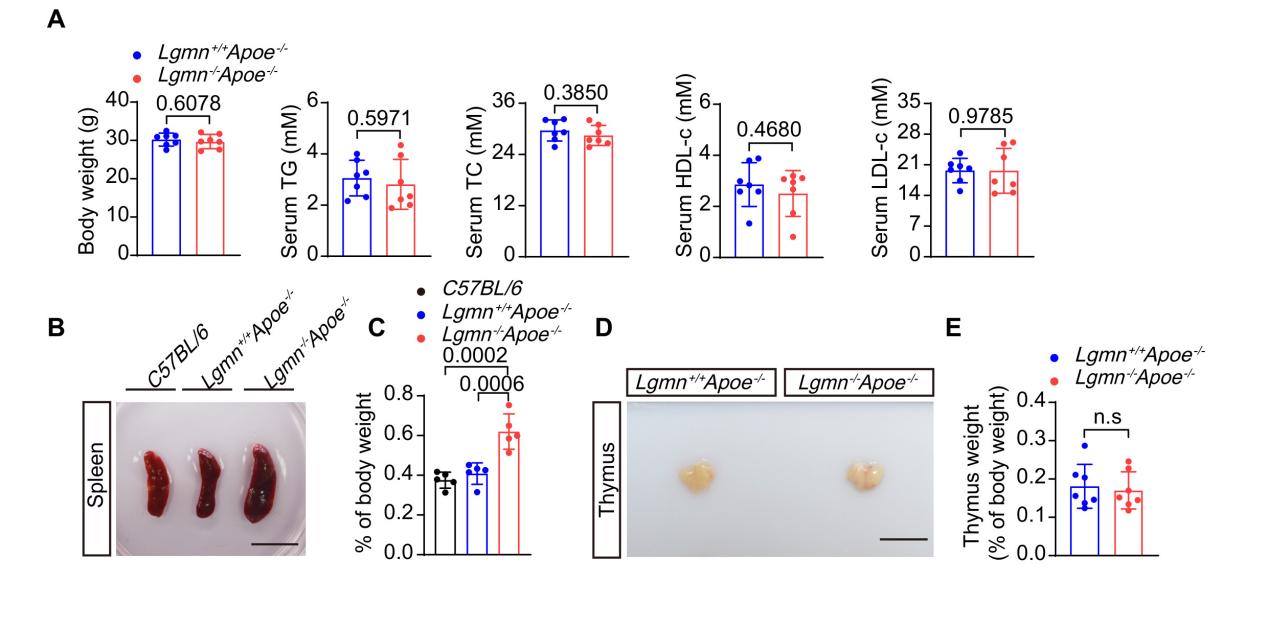
**

**Figure S1. Effect of legumain deletion on lipid levels and thymus weight.**

**A** Body weight, plasma levels of total cholesterol (TC) and triglycerides (TG), high-density lipoprotein cholesterol (HDL-c), and low-density lipoprotein cholesterol (LDL-c) in mice fed a high-fat diet for 12 weeks (n = 7 mice per group).

**B** Representative image of spleens from C57BL/6 mice, *Lgmn^+/+^Apoe^−/−^* mice and *Lgmn^−/−^Apoe^−/−^* mice. Scale bar, 1 cm.

**C** Spleen weight/body weight in C57BL/6 mice, *Lgmn^+/+^Apoe^−/−^* mice and *Lgmn^−/−^Apoe^−/−^* mice (n = 5 mice per group).

**D** Representative images of thymuses from *Lgmn^+/+^Apoe^−/−^* mice and *Lgmn^−/−^Apoe^−/−^* mice. Scale bar, 1 cm.

**E** Thymic weight/body weight in *Lgmn^+/+^Apoe^−/−^* mice and *Lgmn^−/−^Apoe^−/−^* mice (n = 7 mice per group).

TG, triglyceride; TC, total cholesterol; HDL-c, high-density lipoprotein cholesterol; LDL-c, low-density lipoprotein cholesterol.

Data information: The exact *P* value is specified. The *P* value was determined by unpaired two-tailed Student’s *t-*test (A, C and E).

**
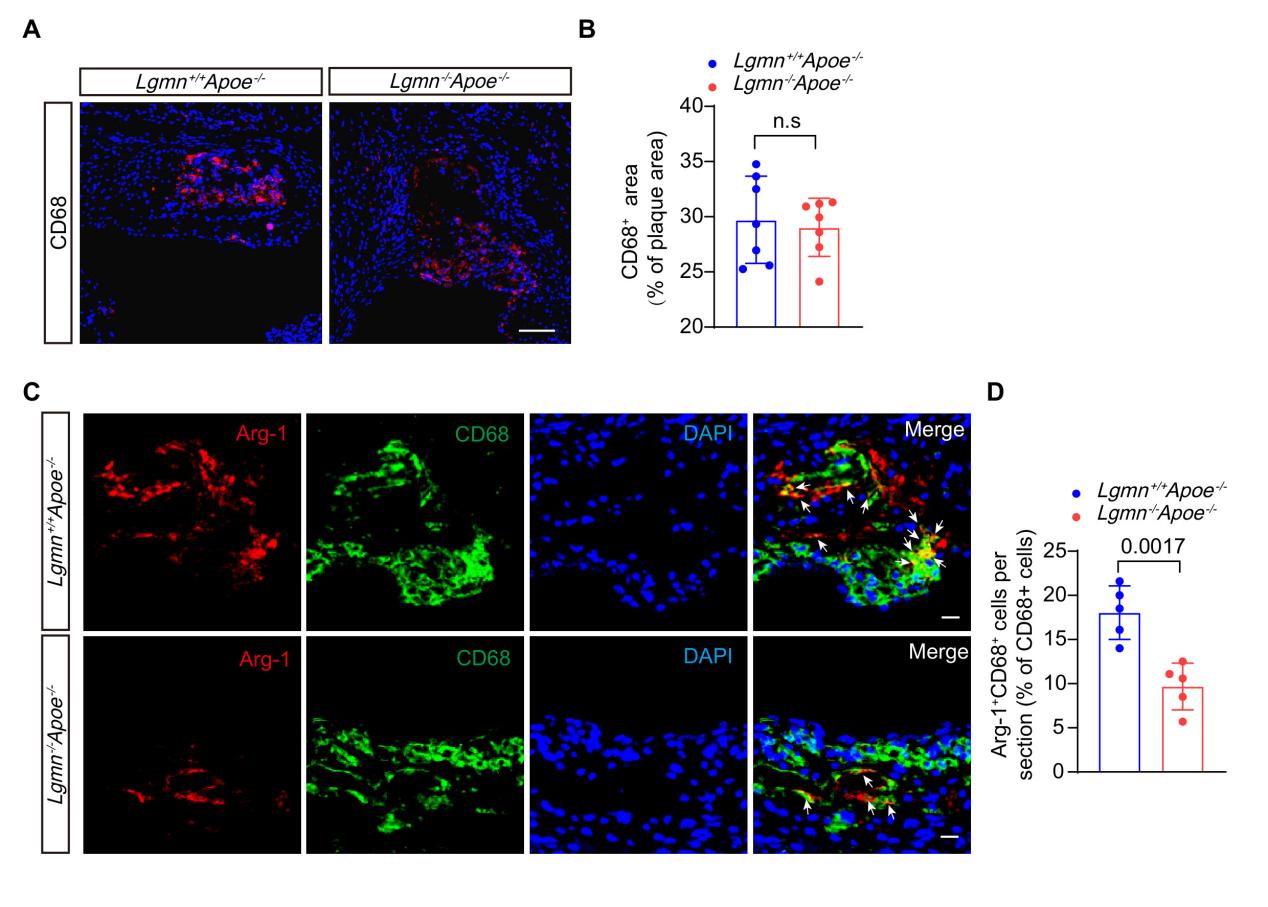
**

**Figure S2. Analysis of macrophage polarization in aortic root plaques.**

**A, B** Representative immunofluorescence images showing CD68^+^ cells in the plaques of *Lgmn^+/+^Apoe^−/−^* mice and *Lgmn^−/−^Apoe^−/−^* mice. Scale bar, 100 µm. The ratio of CD68-positive area to plaque area from each group was quantified (n = 7 mice per group).

**C, D** Representative confocal microscopy images showing immunostaining for arginase 1 (Arg-1, red) and legumain (green) in aortic root plaques from *Lgmn^+/+^Apoe^−/−^* mice and *Lgmn^−/−^Apoe^−/−^* mice. Arrows indicate co-localization of CD68 and Arg-1. Scale bar, 20 µm. The number of Arg-1^+^CD68^+^ cells per section was quantified (n = 5 mice per group).

Arg-1, arginase 1.

Data information: The exact *P* value is specified. The *P* value was determined by unpaired two-tailed Student’s *t-*test (B, D).


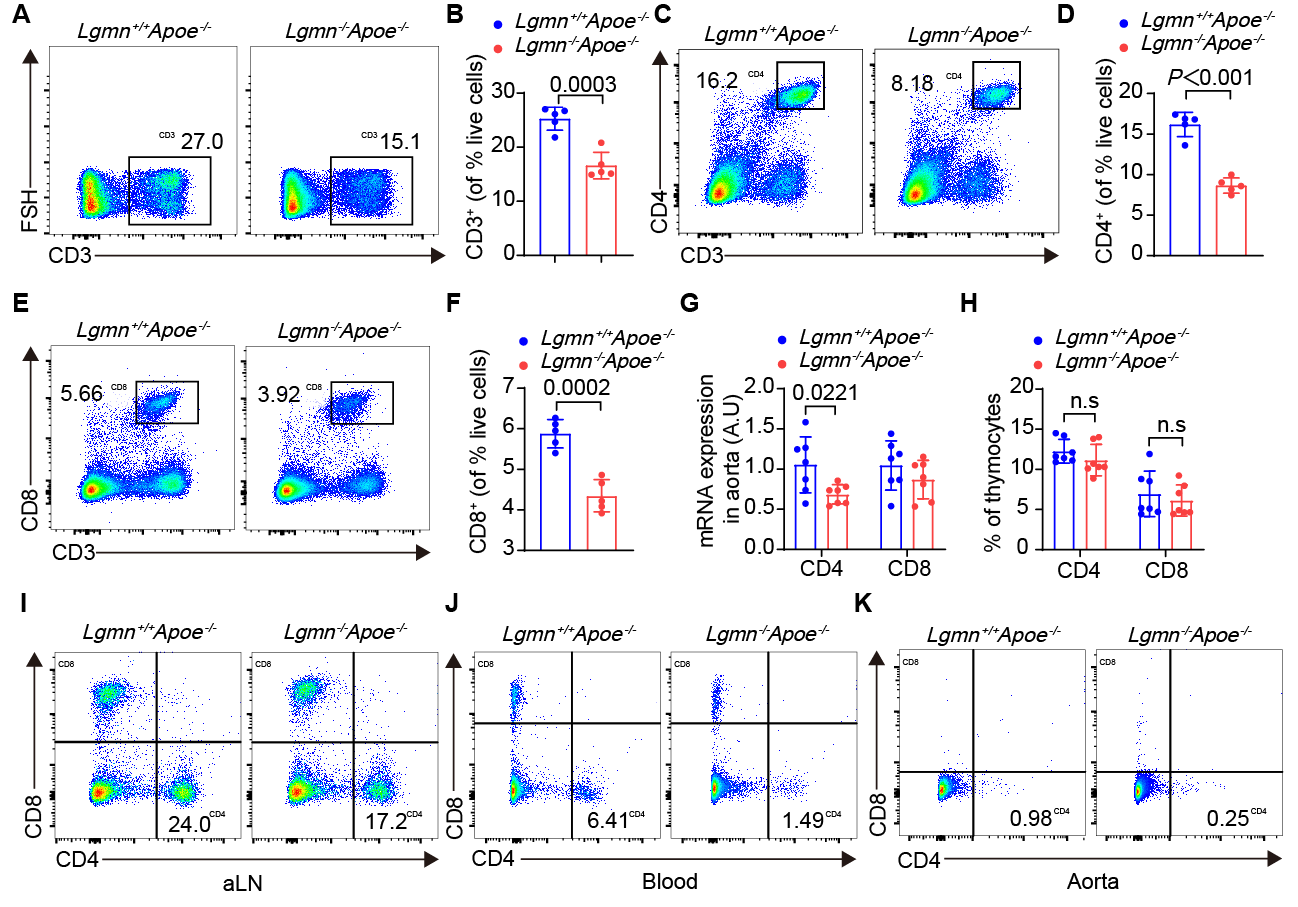


**Figure S3. Legumain deficiency reduces CD4^+^ T cells in the aorta-draining lymph nodes, blood, and aorta.**

**A–F** Representative flow cytometry analysis and quantification of the proportions of CD3^+^ T cells, CD4^+^ T cells, and CD8^+^ T cells among total live splenic cells from *Lgmn^+/+^Apoe^−/−^* mice and *Lgmn^−/−^Apoe^−/−^* mice (n = 5 mice per group).

**G** Expression of CD4 and CD8 was examined at the mRNA level in the aortas of *Lgmn^+/+^Apoe^−/−^* mice and *Lgmn^−/−^Apoe^−/−^* mice (n = 7 mice per group).

**H** The percentage of CD4^+^CD8^−^ (CD4^+^) and CD4^−^CD8^+^ (CD8^+^) T cells in the thymus was quantified (n = 7 mice per group).

**I–K** Flow cytometric analysis of CD4^+^ T cell and CD8^+^ T cell abundance in aorta-draining lymph nodes, blood, and aorta from atherosclerotic *Lgmn^+/+^Apoe^−/−^* mice and *Lgmn^−/−^Apoe^−/−^* mice.

aLN, aorta-draining lymph nodes.

Data information: The exact *P* value is specified. The *P* value was determined by unpaired two-tailed Student’s *t-*test (B, D, F and G) or two-tailed Mann-Whitney U-test (H).


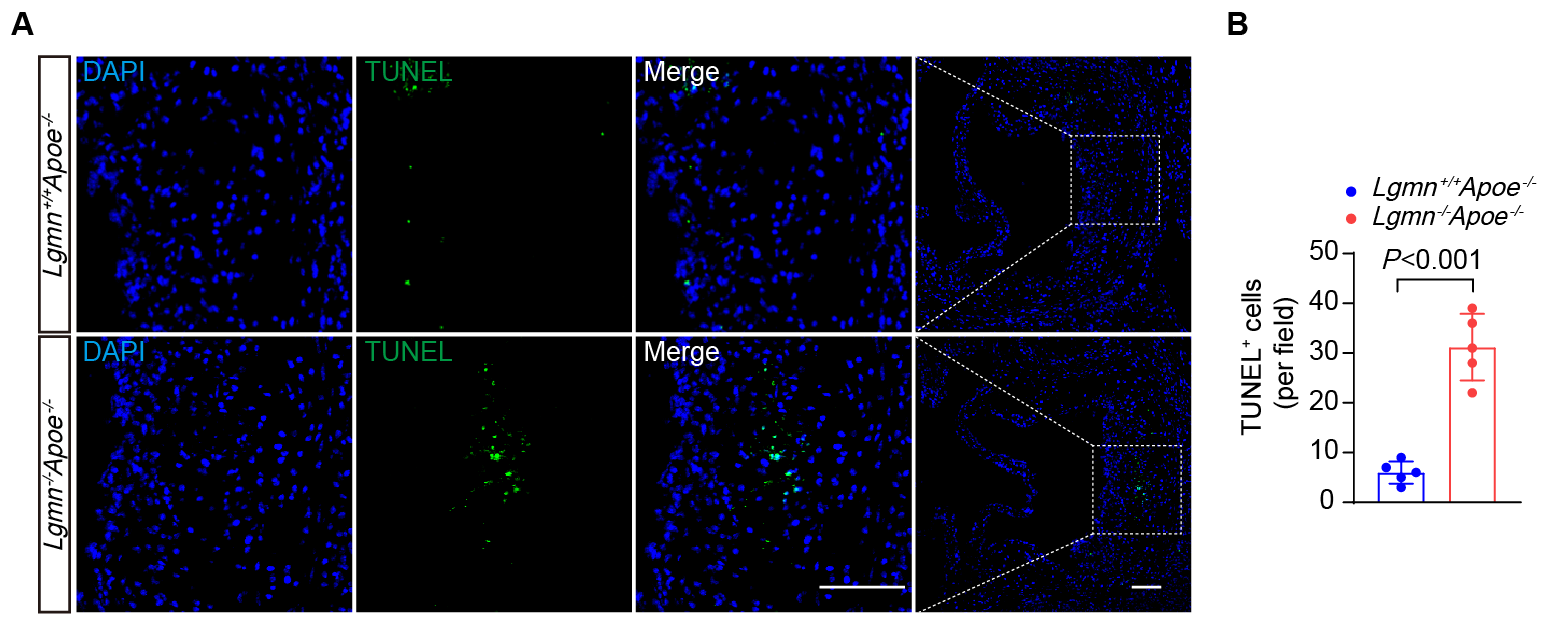
**Figure S4. Analysis of cell apoptosis in atherosclerotic plaques.**

A, B TdT-mediated dUTP nick-end labeling (TUNEL) apoptosis assay. Representative immunofluorescence images showing TUNEL^+^ cells in the plaque of *Lgmn^+/+^Apoe^−/−^* mice and *Lgmn^−/−^Apoe^−/−^* mice. Scale bar, 100 µm. Quantification of the number of TUNEL cells to the total number per field from each group (n = 5 mice per group).

| **Table S1. Complete blood counts in *Lgmn*+/+*Apoe^−/−^* and *Lgmn^−/−^Apoe^−/−^* mice after 12 weeks of high-fat diet (HFD)** | | | |
| --- | --- | --- | --- |
| Parameter | *Lgmn*+/+*Apoe^−/−^*(n=7) | *Lgmn*-/-Apoe-/- (n=7) | *P* |
| Total WBCs, 109/L | 6.846 ± 2.697 | 5.479 ± 3.492 | 0.4283 |
| Lymphocytes, 109/L | 3.520 ± 1.234 | 3.476 ± 2.278 | 0.9647 |
| Monocytes, 109/L | 0.9300 ± 0.6130 | 0.5086 ± 0.4255 | 0.1601 |
| Neutrophils, 109/L | 1.949 ± 0.9809 | 1.244 ± 0.9753 | 0.2028 |
| RBCs, 1012/L | 9.597 ± 1.377 | 6.809 ± 2.543 | 0.0254 |
| Hemoglobin, g/L | 144.1 ± 22.52 | 95.71 ± 46.90 | 0.0299 |
| Platelets, 109/L | 1222 ± 195.5 | 960.4 ± 444.3 | 0.1510 |
| Data are expressed as mean ± SD; WBCs, white blood cells; RBCs, red blood cells. | | | |

| **Table S2. Antibodies** | | | |
| --- | --- | --- | --- |
| Target antigen | Vendor or Source | Catalog No | Working concentration |
| CD3 | Servicebio | GB13014-50 | 1:200 (IF) |
| CD4 | Servicebio | GB15064-100 | 1:200 (IF); 1:400 (IHC) |
| legumain | R & D System | AF2199 | 1:100 (IF) |
| CD68 | Servicebio | GB113109-100 | 1:100 (IF) |
| CD3 | BD Pharmingen | 562600 | 1:100 (FC) |
| CD4 | BD Pharmingen | 552775 | 1:100 (FC) |
| CD8 | BD Pharmingen | 553032 | 1:100 (FC) |
| CD16/CD32 | BD Pharmingen | 553142 | 1:50 (FC) |
| Ki-67 | Biolegend | 652405 | 1:100 (FC) |
| CD44 | Biolegend | 103011 | 1:100 (FC) |
| CD62L | Biolegend | 161203 | 1:100 (FC) |
| CD25 | Biolegend | 102035 | 1:50 (FC) |
| Foxp3 | Biolegend | 126407 | 1:100 (FC) |
| Bcl-2 | Biolegend | 633503 | 1:100 (FC) |
| Alexa Fluor-594 conjugated goat anti-rabbit IgG antibody | Invitrogen | A-21203 | 1:500 (IF) |
| Alexa Fluor-594 conjugated Donkey anti-goat IgG antibody | Invitrogen | A-11058 | 1:500 (IF) |
| IF, Immunofluorscence; IHC:Immunohistochemistry; and FC, Flow cytometry. | | | |
|  | | | |

| **Table S3. QRT-PCR primer sequences** | |
| --- | --- |
| Gene name | Primer Sequence |
| CD28 | Forward: 5'-GTTCTTGGCTCTCAACTTCTTCT-3' |
|  | Reverse: 5'-TGAGGCTGACCTCGTTGCTAT-3' |
| CD40L | Forward: 5'-CCTTGCTGAACTGTGAGGAGA-3' |
|  | Reverse: 5'-CTTCGCTTACAACGTGTGCT-3' |
| Ki-67 | Forward: 5'-ATCATTGACCGCTCCTTTAGGT-3' |
|  | Reverse: 5'-GCTCGCCTTGATGGTTCCT-3' |
| CXCR3 | Forward: 5'-TACGATCAGCGCCTCAATGCCA-3' |
|  | Reverse: 5'-AGCAGGAAACCAGCCACTAGCT-3' |
| CXCL10 | Forward: 5'-CCAAGTGCTGCCGTCATTTTC-3' |
|  | Reverse: 5'-GGCTCGCAGGGATGATTTCAA-3' |
| IFN-γ | Forward: 5'-AGGCCATCAGCAACAACATA-3' |
|  | Reverse: 5'-TGAGCTCATTGAATGCTTGG-3' |
| IL-2 | Forward: 5'-TGAGCAGGATGGAGAATTACAGG-3' |
|  | Reverse: 5'-GTCCAAGTTCATCTTCTAGGCAC-3' |
| BIM | Forward: 5'-GGAGATACGGATTGCACAGGAG-3' |
|  | Reverse: 5'-CTCCATACCAGACGGAAGATAAAG-3' |
| BAX | Forward: 5'-AGGATGCGTCCACCAAGAAGCT-3' |
|  | Reverse: 5'-TCCGTGTCCACGTCAGCAATCA-3' |
| BAK | Forward: 5'-GGAATGCCTACGAACTCTTCACC-3' |
|  | Reverse: 5'-CAAACCACGCTGGTAGACGTAC-3' |
| Bcl-2 | Forward: 5'-CCTGTGGATGACTGAGTACCTG-3' |
|  | Reverse: 5'-AGCCAGGAGAAATCAAACAGAGG-3' |
| Bcl-XL | Forward: 5'-GCCACCTATCTGAATGACCACC-3' |
|  | Reverse: 5'-AGGAACCAGCGGTTGAAGCGC-3' |
| CD4 | Forward: 5'-CGAACATCTGTGAAGGCAAA-3' |
|  | Reverse: 5'-GAGCTCTTGTTGGTTGGGAA-3' |
| CD8 | Forward: 5'-ACGGCCCTTTCTCAGTATCA-3' |
|  | Reverse: 5'-GTTGGGGCAGTTGTAGGAAG-3' |
| GAPDH | Forward: 5'-GGTTGTCTCCTGCGACTTCA-3' |
|  | Reverse: 5'-TGGTCCAGGGTTTCTTACTCC-3' |
