# Supplementary figures and images for: Legumain deficiency halts atherogenesis by modulating T cell receptor signaling
Source: Aging Cell. 2024 Oct 29;24(2):e14391. doi: 10.1111/acel.14391 (PMC11822642; doi:10.1111/acel.14391)

**A**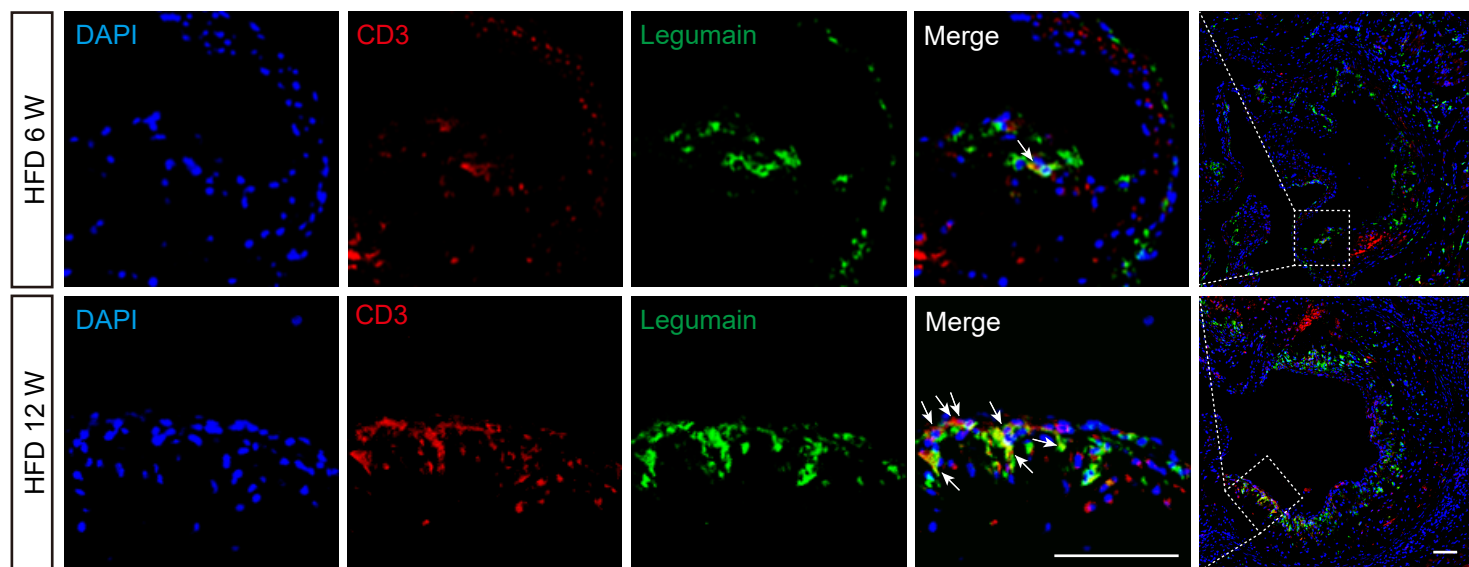**B**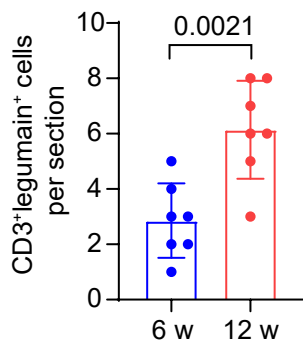**C**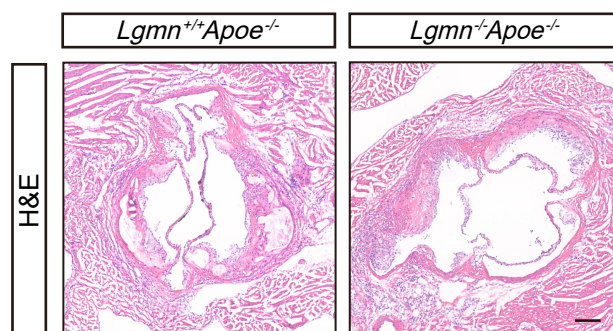**D**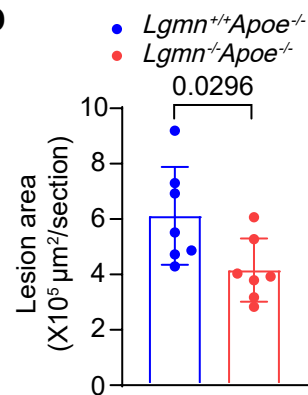**E**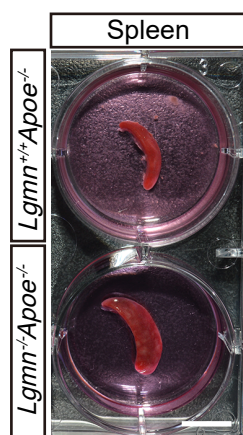**F**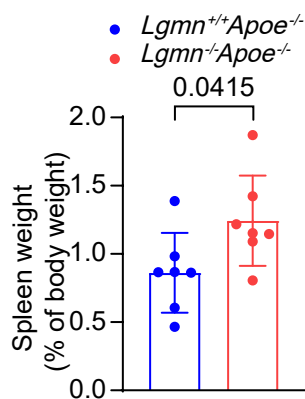**G**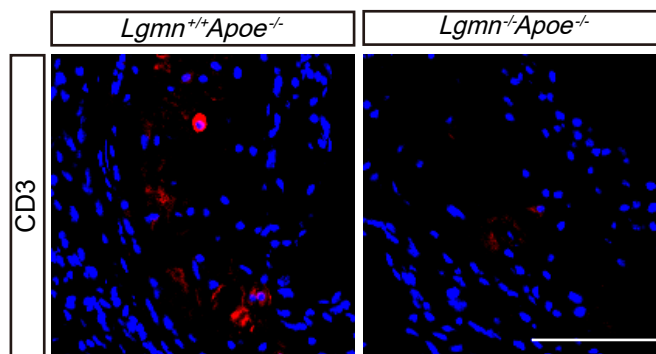**H**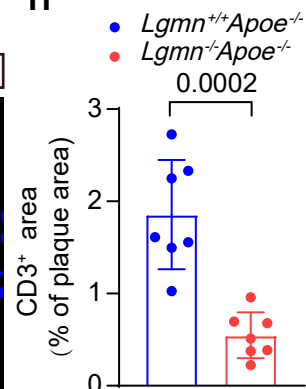

Supplement: Supplementary file 1 — Appendix S1. [file ACEL-24-e14391-s001.zip › Appendix S1/Supplementary Material/Figure_1.pdf]

**A**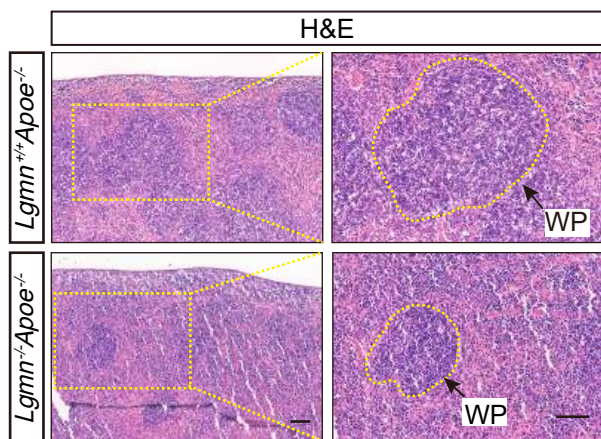**B**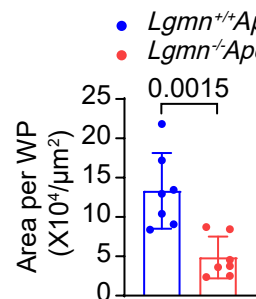**C**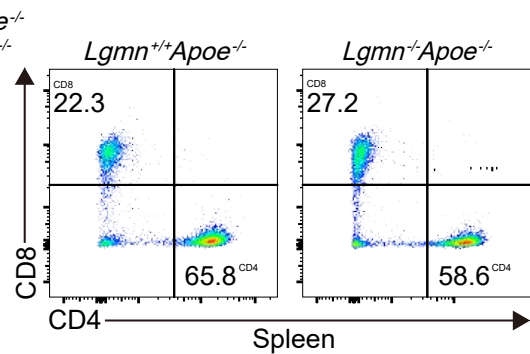**D**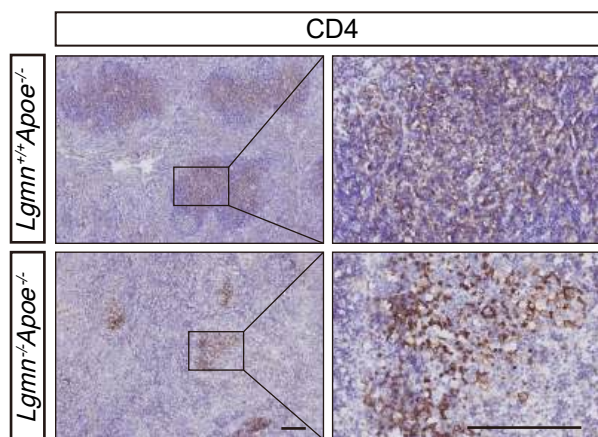**E**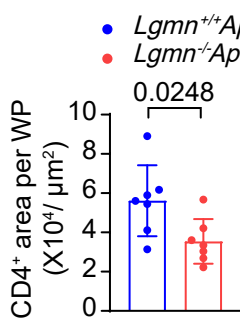**F**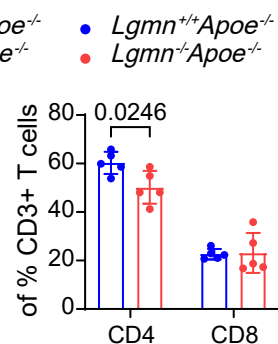**G**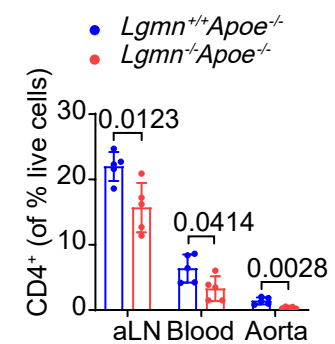**H**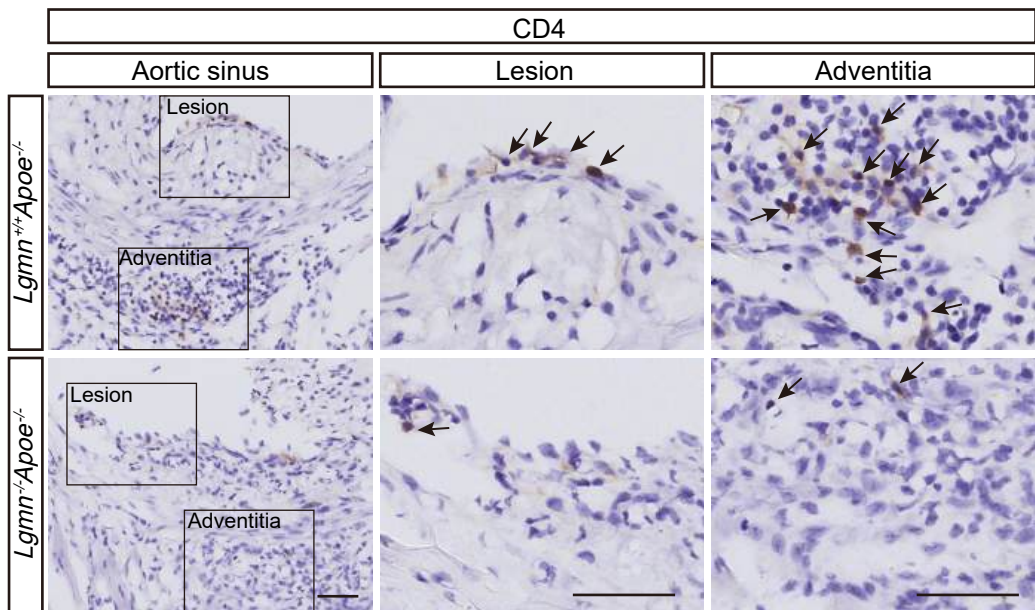**I**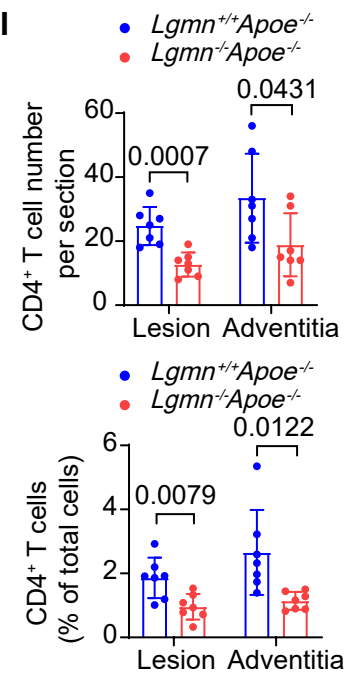

Supplement: Supplementary file 1 — Appendix S1. [file ACEL-24-e14391-s001.zip › Appendix S1/Supplementary Material/Figure_2.pdf]

**A**

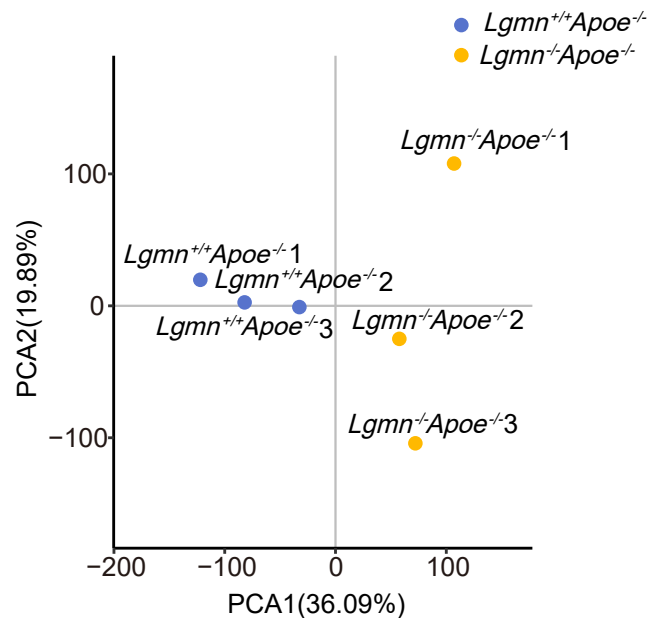

**C**

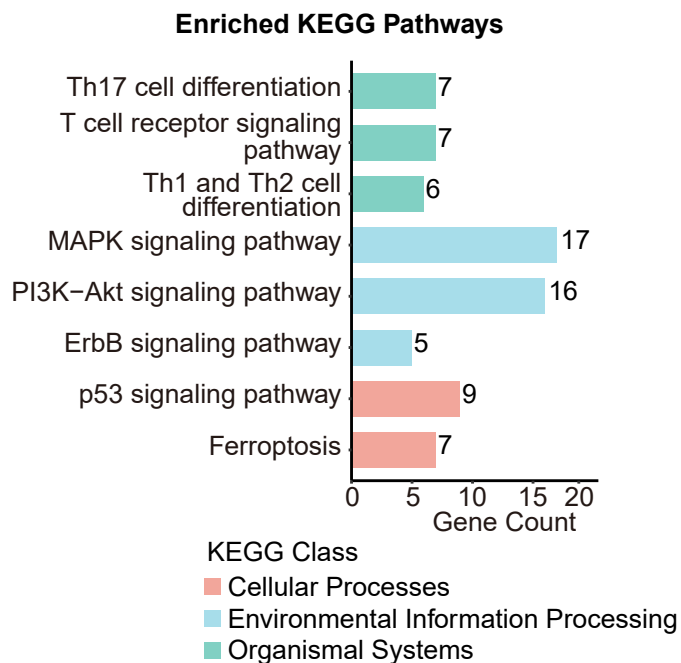

# E

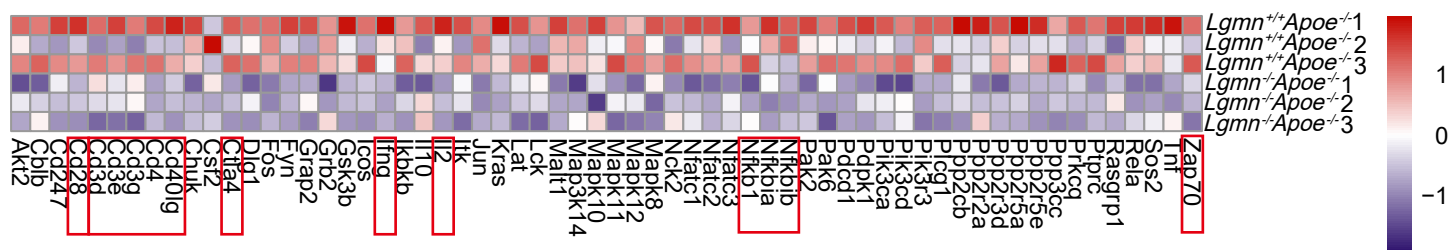

# B

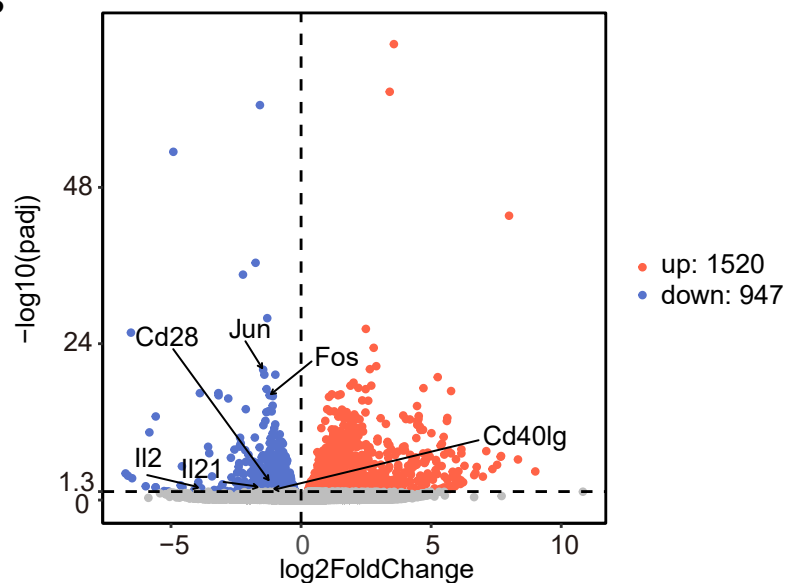

**D**

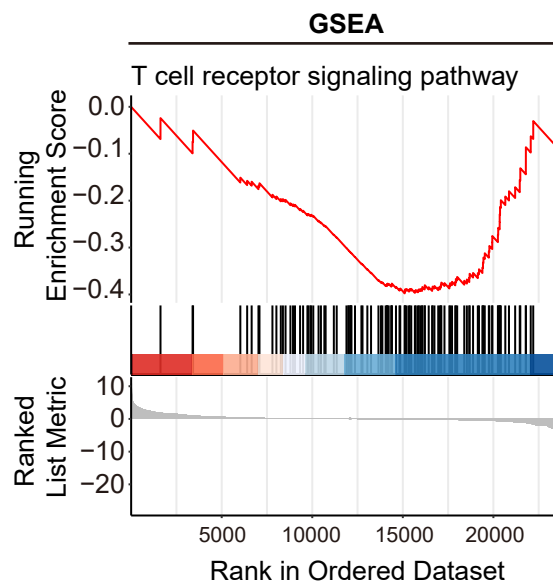

Supplement: Supplementary file 1 — Appendix S1. [file ACEL-24-e14391-s001.zip › Appendix S1/Supplementary Material/Figure_3.pdf]

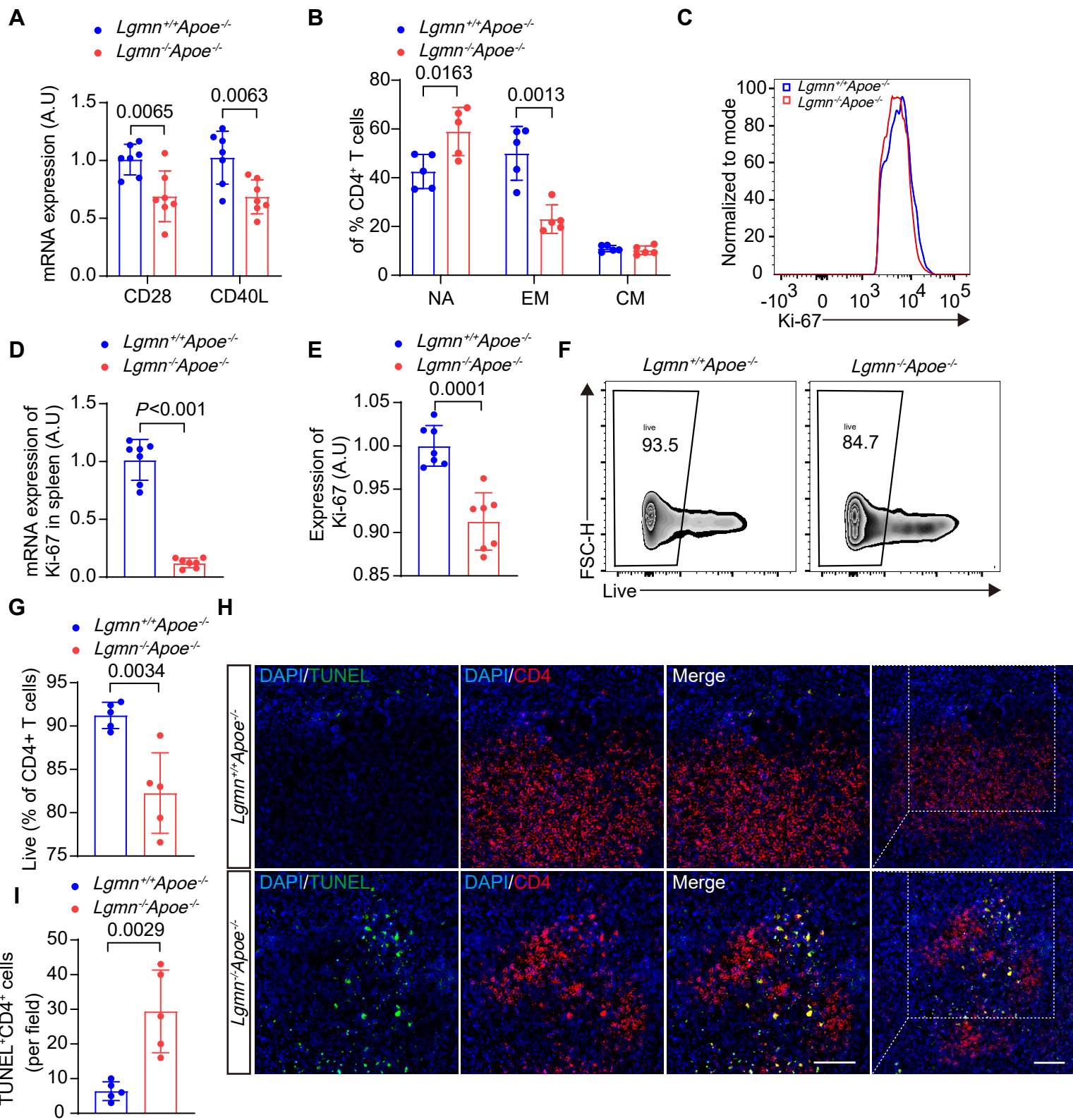

Supplement: Supplementary file 1 — Appendix S1. [file ACEL-24-e14391-s001.zip › Appendix S1/Supplementary Material/Figure_4.pdf]

**A**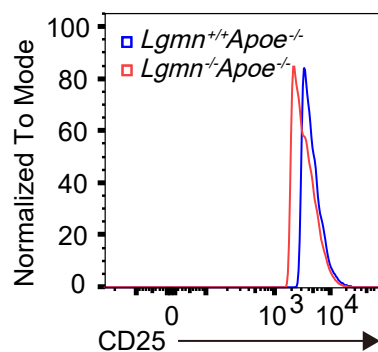**B**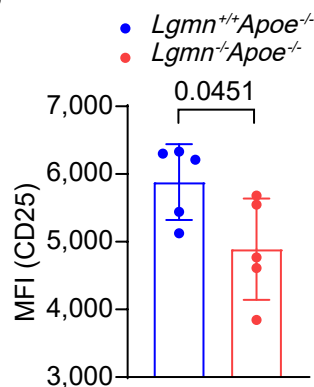**C**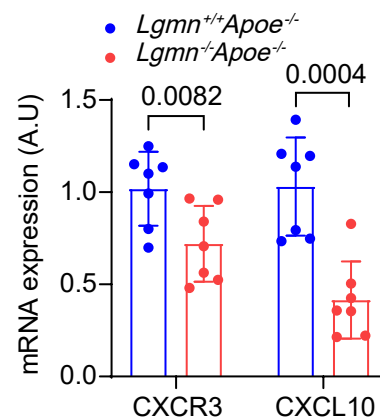**D**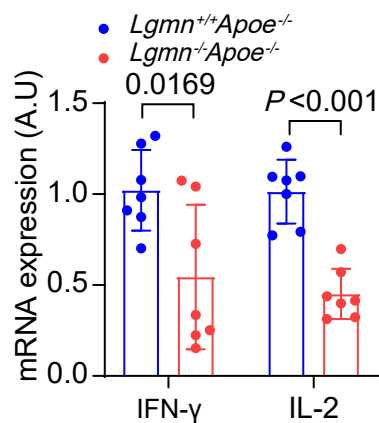**E**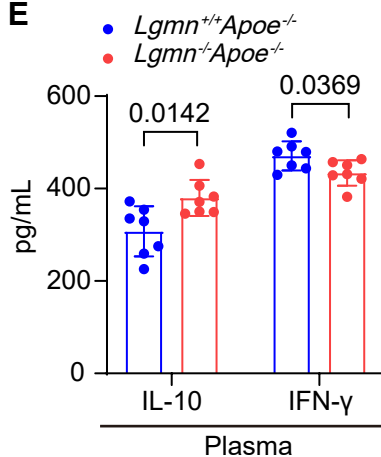**F**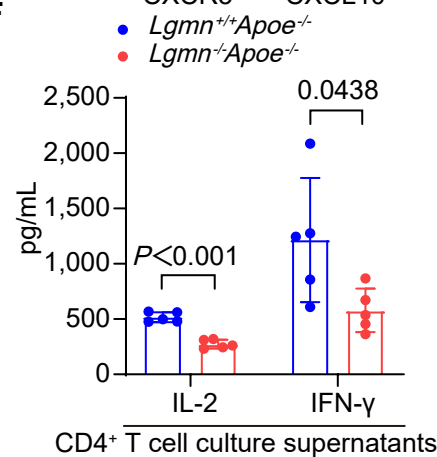**G**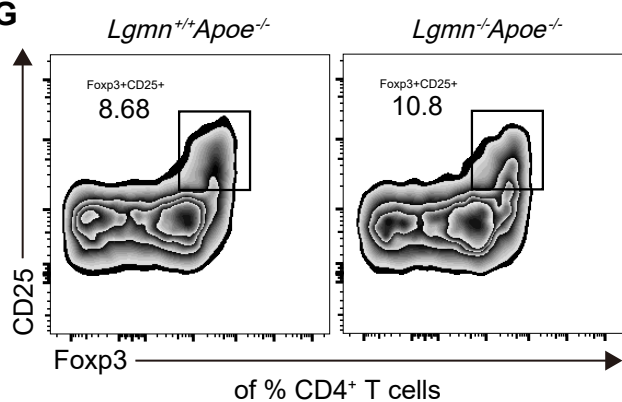**H**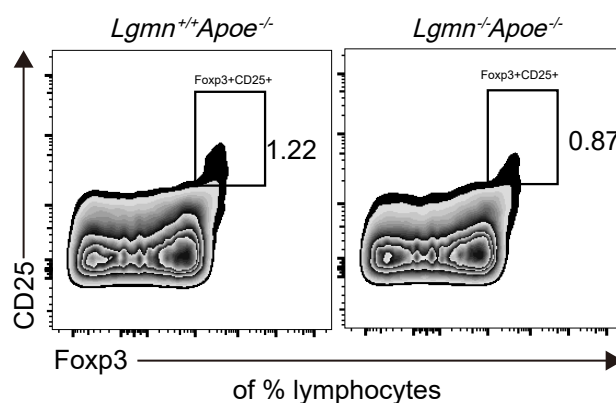**I**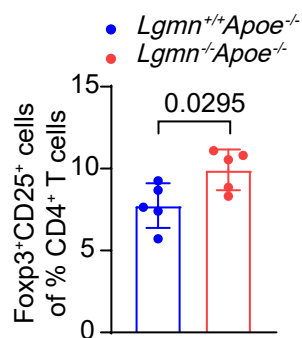**J**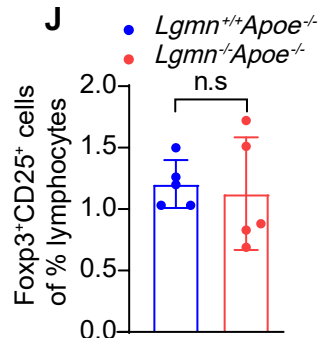

Supplement: Supplementary file 1 — Appendix S1. [file ACEL-24-e14391-s001.zip › Appendix S1/Supplementary Material/Figure_5.pdf]

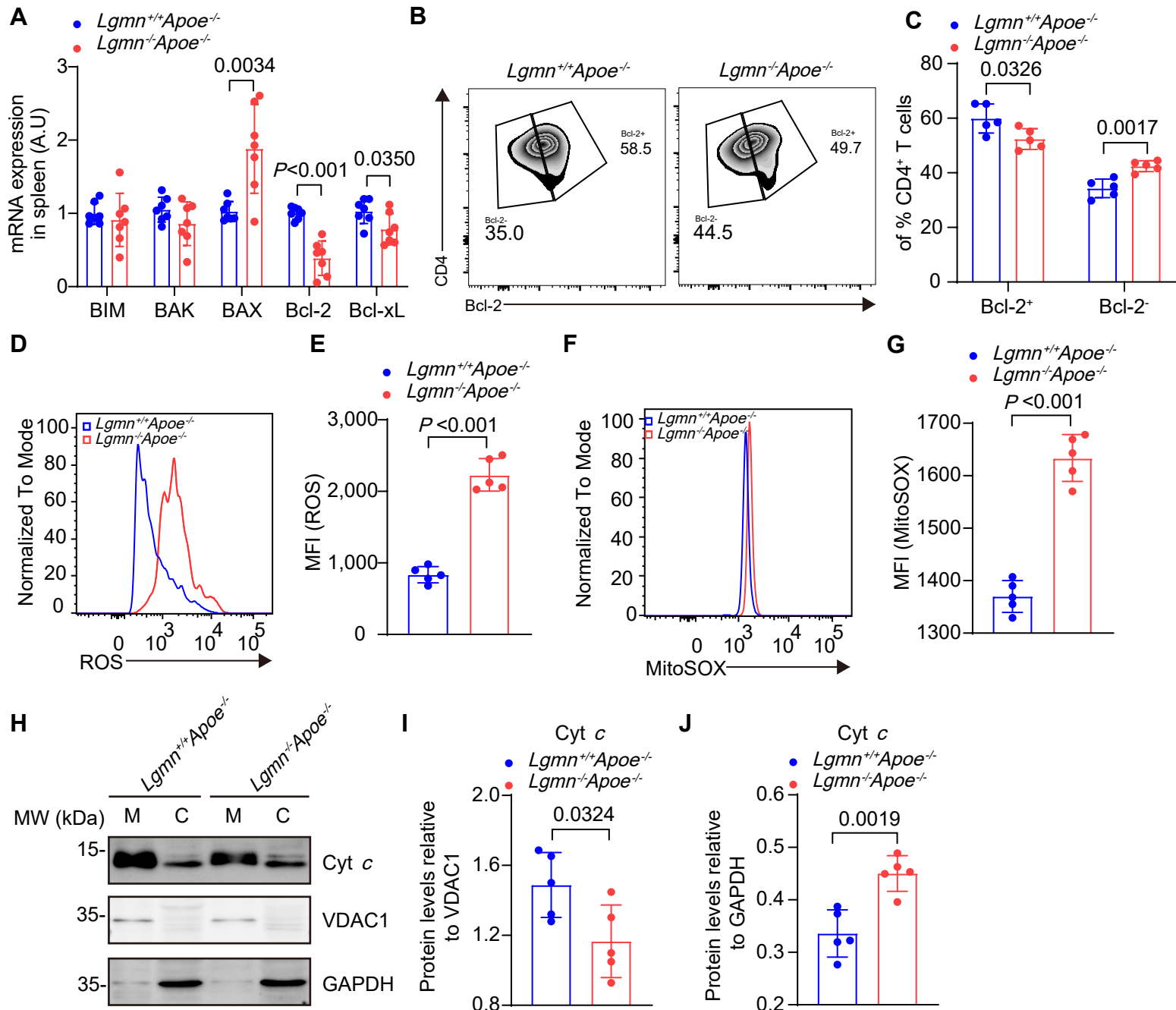

Supplement: Supplementary file 1 — Appendix S1. [file ACEL-24-e14391-s001.zip › Appendix S1/Supplementary Material/Figure_6.pdf]

**A**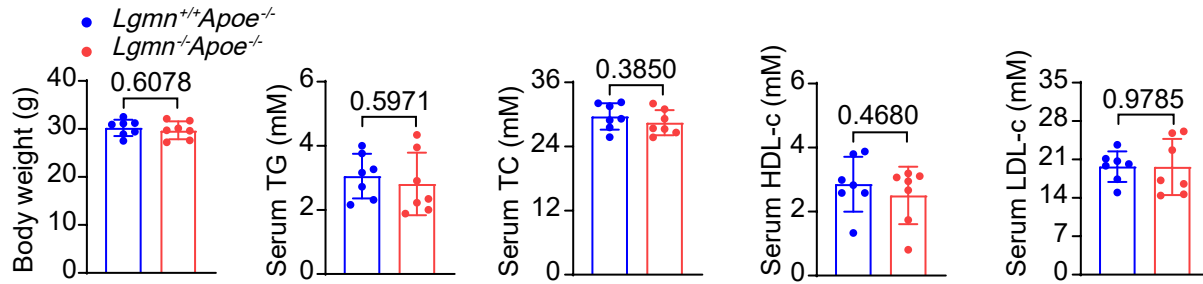**B**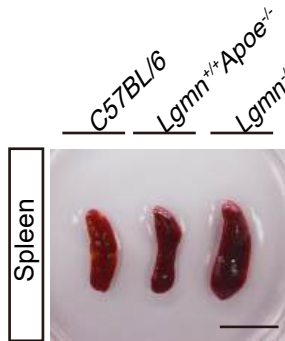**C**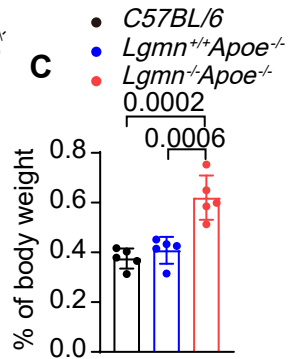**D**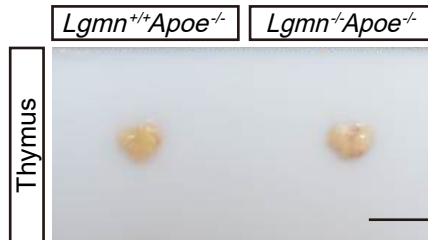**E**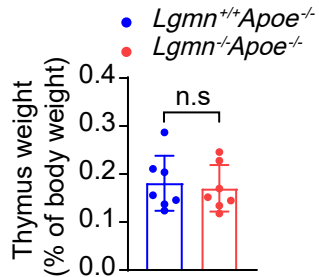

Supplement: Supplementary file 1 — Appendix S1. [file ACEL-24-e14391-s001.zip › Appendix S1/Supplementary Material/Figure_S1.pdf]

**A**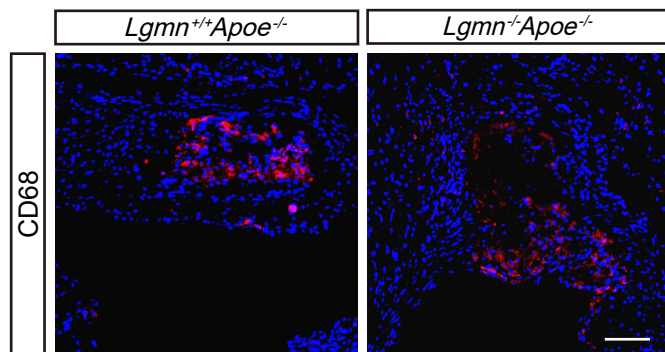**B**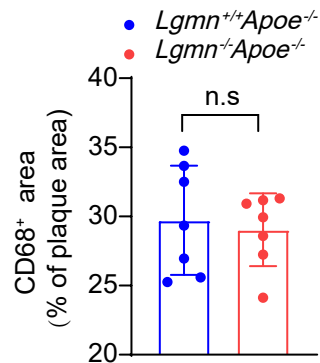**C**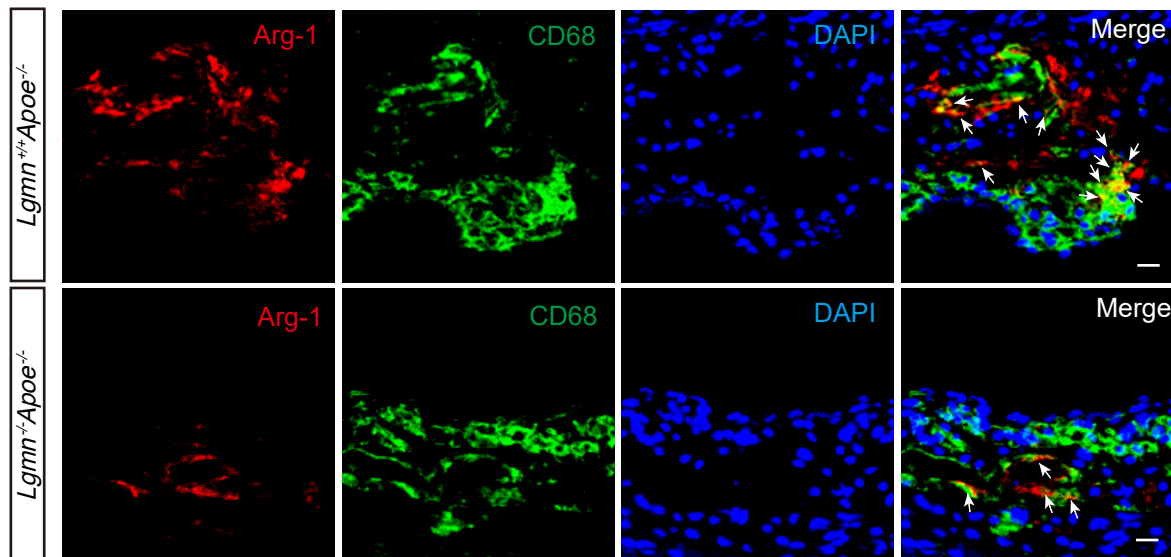**D**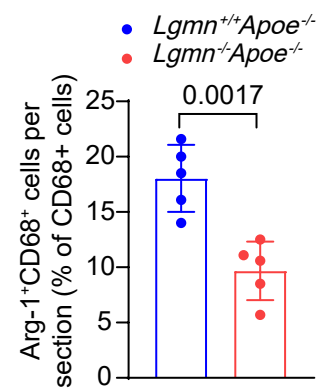

Supplement: Supplementary file 1 — Appendix S1. [file ACEL-24-e14391-s001.zip › Appendix S1/Supplementary Material/Figure_S2.pdf]

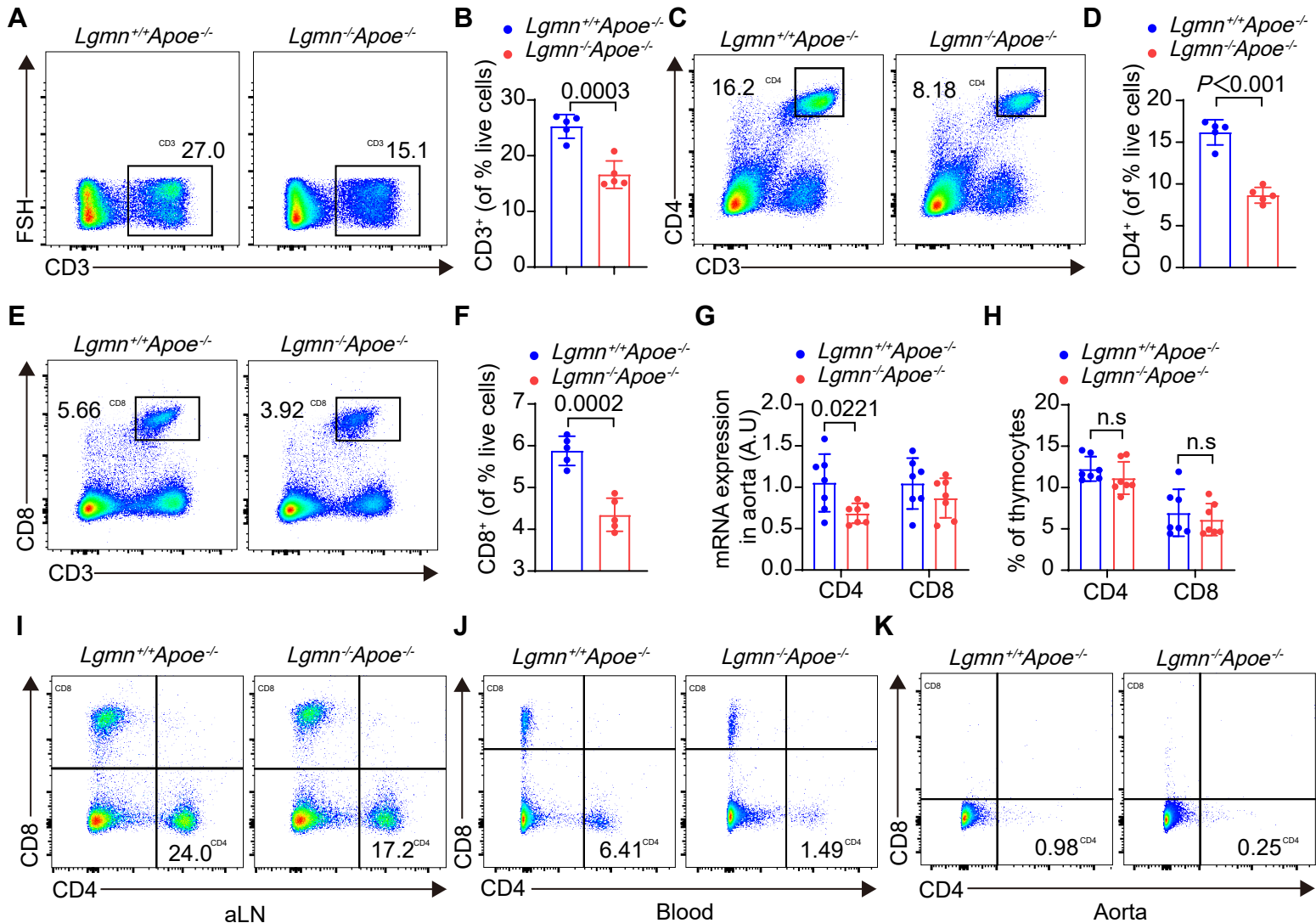

Supplement: Supplementary file 1 — Appendix S1. [file ACEL-24-e14391-s001.zip › Appendix S1/Supplementary Material/Figure_S3.pdf]

**A**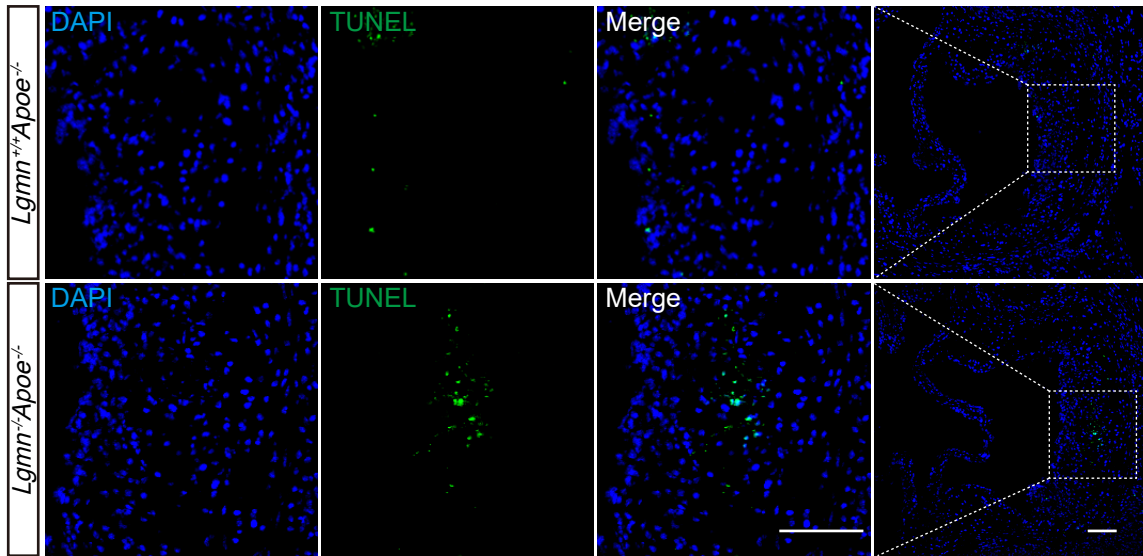**B**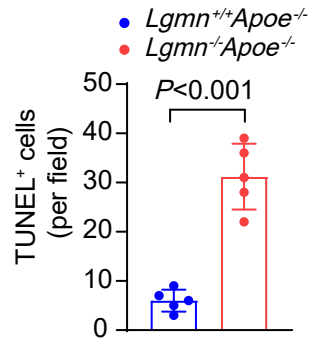

Supplement: Supplementary file 1 — Appendix S1. [file ACEL-24-e14391-s001.zip › Appendix S1/Supplementary Material/Figure_S4.pdf]
